# Ewing’s Sarcoma: An Analysis of miRNA Expression Profiles and Target Genes in Paraffin-Embedded Primary Tumor Tissue

**DOI:** 10.3390/ijms17050656

**Published:** 2016-04-30

**Authors:** Antonina Parafioriti, Caterina Bason, Elisabetta Armiraglio, Lucia Calciano, Primo Andrea Daolio, Martina Berardocco, Andrea Di Bernardo, Alessia Colosimo, Roberto Luksch, Anna C. Berardi

**Affiliations:** 1 Unità Operativa Complessa (U.O.C.) Azienda Socio Sanitaria Territoriale Centro Specialistico Ortopedico Traumatologico Gaetano Pini-CTO, Milano 20122, Italy; elisabetta.armiraglio@asst-pini-cto.it (E.A.); andrea.dibernardo@asst-pini-cto.it (A.D.B.); 2 Dipartimento di Medicina, Sezione di Medicina Interna B, Università di Verona, Verona 37134, Italy; caterina.bason@univr.it; 3 Dipartimento di Sanità Pubblica e Medicina di Comunità, Sezione di Epidemiologia e Statistica Medica, Università di Verona, Verona 37134, Italy; lucia.calciano@univr.it; 4 Unità Operativa Complessa (U.O.C.) Chirurgia Ortopedica Oncologica, Azienda Socio Sanitaria Territoriale Centro Specialistico Ortopedico Traumatologico Gaetano Pini-CTO, Milano 20122, Italy; PrimoAndrea.Daolio@asst-pini-cto.it; 5 Unità Operativa Complessa (U.O.C.) Immunoematologia-Medicina Trasfusionale e Laboratorio di Ematologia, Laboratorio di Ricerca “Cellule Staminali” Azienda Unità Sanitaria Locale (AUSL)-Ospedale Santo Spirito, Pescara 65125, Italy; martina.berardocco@gmail.com; 6 Facoltà di Medicina Veterinaria, Università di Teramo, Teramo 64100, Italy; acolosimo@unite.it; 7 Dipartimento di Oncologia Pediatrica, Fondazione-Istituto di Ricovero e Cura a Carattere Scientifico-(IRCCS) Istituto Nazionale dei Tumori, Milano 20133, Italy; Roberto.Luksch@istitutotumori.mi.it

**Keywords:** Ewing’s Sarcoma, microRNAs, human mesenchymal stem cells, miRTarBase database

## Abstract

The molecular mechanism responsible for Ewing’s Sarcoma (ES) remains largely unknown. MicroRNAs (miRNAs), a class of small non-coding RNAs able to regulate gene expression, are deregulated in tumors and may serve as a tool for diagnosis and prediction. However, the status of miRNAs in ES has not yet been thoroughly investigated. This study compared global miRNAs expression in paraffin-embedded tumor tissue samples from 20 ES patients, affected by primary untreated tumors, with miRNAs expressed in normal human mesenchymal stromal cells (MSCs) by microarray analysis. A miRTarBase database was used to identify the predicted target genes for differentially expressed miRNAs. The miRNAs microarray analysis revealed distinct patterns of miRNAs expression between ES samples and normal MSCs. 58 of the 954 analyzed miRNAs were significantly differentially expressed in ES samples compared to MSCs. Moreover, the qRT-PCR analysis carried out on three selected miRNAs showed that miR-181b, miR-1915 and miR-1275 were significantly aberrantly regulated, confirming the microarray results. Bio-database analysis identified *BCL-2* as a bona fide target gene of the miR-21, miR-181a, miR-181b, miR-29a, miR-29b, miR-497, miR-195, miR-let-7a, miR-34a and miR-1915. Using paraffin-embedded tissues from ES patients, this study has identified several potential target miRNAs and one gene that might be considered a novel critical biomarker for ES pathogenesis.

## 1. Introduction

Ewing’s Sarcoma (ES) is a highly aggressive bone and soft tissue tumor that mainly affects children and young adults. This tumor is characterized by the unique chromosomal translocation t(11;22)(q24;q12) (which fuses the *EWS* gene on chromosome 22 with the *FLI-1* gene on chromosome 11) leading to a fusion protein which is composed of EWS (Ewing Sarcoma protein) (or rarely, liposarcoma/fused in sarcoma protein) (FUS/TLS) and a member of the Ets transcription factor family [1]. In 85%–90% of cases, this translocation fuses the 5′ end of the *EWS* gene to the 3′ end of the *FLI-1* gene, giving rise to the EWS-FLI-1 fusion protein, in which sequences containing the potent *EWS* transactivation domain are joined to sequences containing the DNA-binding domain (DBD) of *FLI-1* [2]. This EWS/FLI-1 fusion oncoprotein is responsible for the transcriptional deregulation of target genes, such as the CD99 membrane receptor [1]. CD99 altered expression contributes to the Ewing’s tumor oncogenesis by modulating the growth and differentiation of tumor cells. Currently the precise cellular origin of ES is still under debate. Primitive neural crest cells, hematopoietic cells, muscle cells and mesenchymal stromal cells (MSCs) have all been considered as possible cellular source of this type of sarcoma [3]. A growing body of literature supports the mesenchymal origin of ES [4]. Recent genomic studies have identified MSCs as the most closely related normal tissue and the most convincing candidate tissue to explain the cellular origin of ES [5]. In addition, a recent research carried out into the sarcoma microenvironment indicates that MSCs could play an active part in the generation of supportive stromas [6]. Thus, MSCs are an excellent molecular tool to investigate oncogenesis in ES.

Although most patients with localized ES can be cured with intensive therapy, clinical evolution varies largely amongst patients. Unfortunately, little is known about the biological features that distinguish low-risk from high-risk disease, or about the mechanisms of ES progression. Indeed, histological response after preoperative chemotherapy remains a significant indicator of prognosis.

Moreover, recent reports showed that evasion of apoptosis could be a feature of ES similar to that observed in several different cancer cells. It has been demonstrated that *BCL2* is involved in this pathway, by inhibiting cell apoptosis and enhancing chemoresistance [7]. *BCL2* is a proto-oncogene which, under normal conditions, binds the pro-apoptotic proteins (such as BAX, BAK, PUMA), impairing their activity and maintaining mitochondrial integrity and survival of the cells. In the presence of DNA damage or cytotoxic stimuli (such as chemotherapy or radiotherapy) [8], the expression of *BCL2* is inhibited and the activity of the target pro-apoptotic proteins is increased. Although deregulated *BCL2* expression is critical for apoptosis, which is a key step in tumorigenesis, it is not clear the mechanism underlying the stability of the BCL2 protein. Further studies aimed at understanding these mechanisms might contribute to cancer therapy.

In this context, while the role of aberrantly expressed miRNAs is well established for other types of cancer, few studies exist for ES. MiRNAs are a class of 19–25-nucleotide non-coding RNAs, possessing critical roles in the regulation of gene expression in normal and pathological tissues. Moreover, they are frequently unregulated in cases of cancer, with potentially severe biological consequences. However, relatively limited knowledge is available regarding the role of miRNAs in pediatric cancers, including ES. In fact, the biological processes or the mechanisms underlying aberrant miRNAs expression in ES are not fully understood [8]. Growing evidence indicates that miRNAs play a crucial role in the post-transcriptional regulation of several genes which play a role in multiple biological functions (e.g., proliferation, differentiation, apoptosis, metabolism, angiogenesis and stress response) [9,10,11]. As a consequence, their abnormal expression, caused by chromosomal alterations, might contribute to develop cancer and/or its progression [11,12]. Moreover, different types of cancer have distinct miRNAs profiles that can be used as molecular biomarkers for tumor diagnosis, prognosis and the prediction of therapeutic responses. The class of non-coding RNAs with tumor-suppressive and oncogenic functions thus broadens to include the family of miRNAs. In addition to their unique high stability, their specific association with cancers makes miRNAs promising biomarkers for various diseases in humans [11,13]. Many databases have been developed to predict miRNAs’ target genes. The easily accessed miRTarBase serves as a repository for experimentally validated miRNAs’ target interaction [14]. The EWS/FLI-1 fusion protein and the homogeneity of ES biopsies are important elements in order to establish a list of biological biomarkers for practical and clinical use.

This study aims at the identification of miRNAs that might be relevant for the understanding of the oncogenic mechanism in ES. For this reason, the miRNAs expression patterns of 20 primary ES tumors were examined by microarray analysis and compared with the miRNAs expression patterns of MSCs commercial lines from 4 normal donors, used as controls. The results showed the identification of 58 significantly deregulated miRNAs, 10 of which are present in the majority of our samples. Moreover, we have identified one target gene that could represent a novel biomarker for understanding the pathogenesis of ES.

## 2. Results

### 2.1. Clinical Features of Ewing’s Sarcoma (ES) Patients

The diagnosis of ES in the 20 subjects was established on the basis of clinical and morphological histology, routine immunohistochemistry and using molecular diagnostic techniques. Representative data are shown in Table 1.

**Figure 1:**
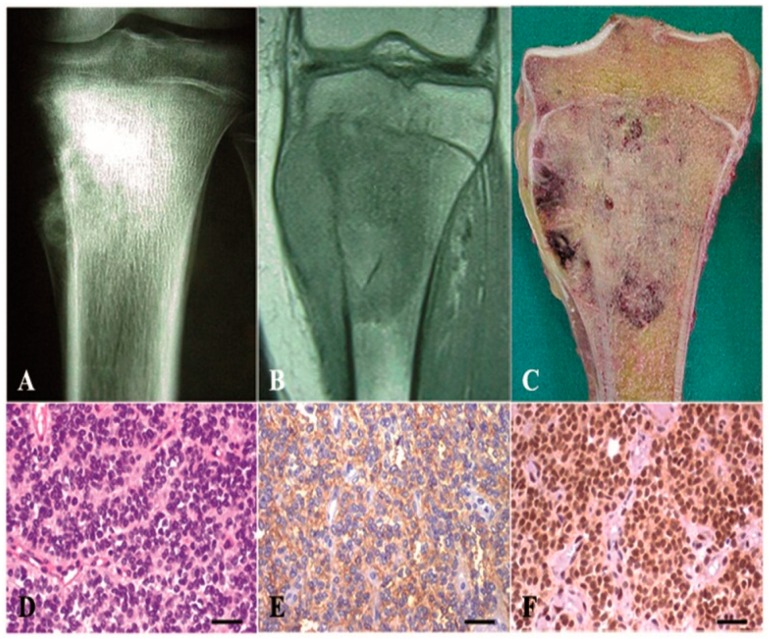
Ewing sarcoma of the left proximal tibia in a 14-year-old girl. (**A**) Plain radiograph showing lytic neoplasia with massive medullary bone involvement, cortical destruction and periosteal reaction with bone formation and soft-tissue mass; (**B**) Coronal T1-weighted MRI revealing significant involvement of metaphysis and an extraosseous tumoral component; (**C**) Macroscopic appearance showing dominant solid architecture: the tumor permeates the medullary bone, infiltrating the cortex, has destructive borders and there is an evident soft-tissue mass; (**D**) Uniform, small, round neoplastic cells with round nuclei containing fine chromatin and scanty clear/eosinophilic cytoplasm (H,E); (**E**) Classical immunohistochemical membranous positivity for CD99; (**F**) Strong nuclear immunoreactivity for FLI-1; (20×).

Immunohistochemical markers were used in the ES routine diagnostic setting as a standard procedure. CD99 and FLI-1 remain the most widely recognized markers for ES. Consequently, only patients who were positive for over-expression of the transmembrane glycoprotein CD99 and also displayed EWS-FLI-1 fusion were selected (Figure 1, Table 1). Figure 1 also shows the typical morphology of ES cells (small, round tumor-cells).

### 2.2. Expression Profiling of miRNAs in Ewing’s Sarcoma Tumors

**Figure 2:**
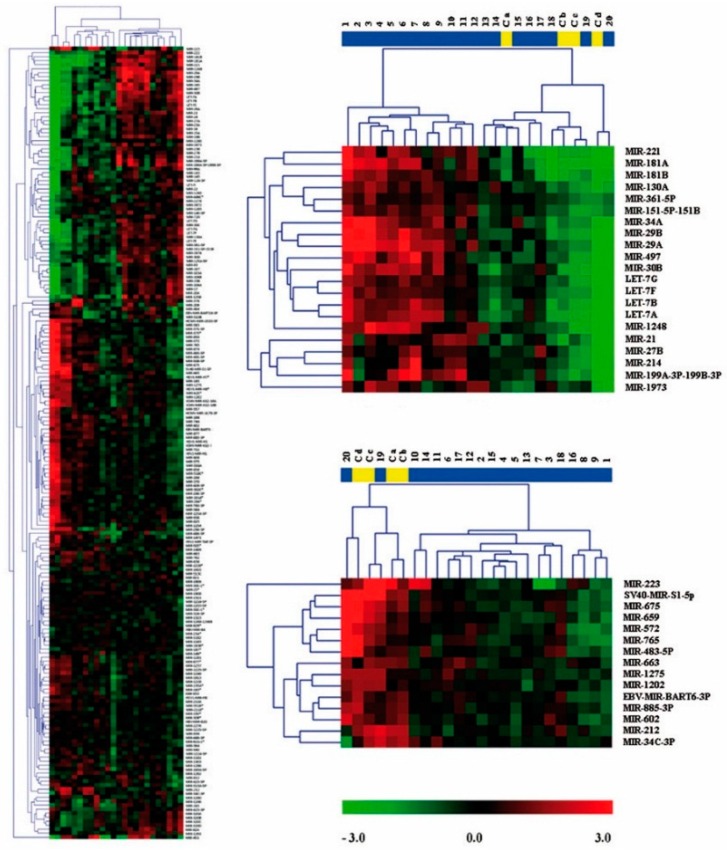
Global miRNAs expression and unsupervised hierarchical clustering of ES tumors and normal MSCs using 3D-Gene miRNA oligo chips. A partial heat-map depicts the distinct patterns of miRNAs expression in the samples. Vertical columns and horizontal rows represent individual samples and miRNAs, respectively. The red or green color represents relatively high or low expression, respectively. An overall expression pattern of 954 miRNAs is shown by a compressed heat-map (left).

Archived formalin-fixed paraffin-embedded (FFPE) tissue samples represent excellent resources for biomarker discovery. In order to identify clusters of miRNAs which may be involved in the oncogenesis of ES, a microarray technology approach was used to investigate global miRNAs expression patterns. In this regard, we analyzed the expression of 954 miRNAs and also compared miRNA levels in 20 ES samples and in MSCs lines from 4 normal donors (presumed cells of ES origin) (Table S1). Hierarchical unsupervised cluster analysis based on the expression of these 954 miRNAs, all with valid duplicate spots, displayed distinct expression profiles for each sample type. The majority of the samples tested were correctly clustered by sample and by miRNAs (Figure 2).

MiRNAs expression levels were calculated relatively to invariably express nuclear RNA U6. To select significant miRNAs (*i.e.*, *p* < 0.05) ΔΔ*C*t and fold change (FC) were calculated for test comparisons (the 20 ES patients were compared to the four MSCs controls). MiRNAs that displayed a FC ≥1.2 or ≤−0.5 were considered to be differentially expressed. Using this criteria we found 366 deregulated miRNAs. The 366 *p*-values were corrected for controlling the false discovery rate (FDR) by using the Simes multiple-test procedure [14]. We found 58 significantly deregulated miRNAs (uncorrected *p* < 0.05), but none of these were significant after adjusting for multiple testing. The FDR-corrected cut-off for statistical significance is equal to 0.00014. We found weak departures from normality for a few miRNAs. Therefore, in order to improve uniformity of the results we used the exact Mann-Whitney *U* test for all comparisons. Among the 58 observed miRNAs which were considered for evaluation, we found that 36 were up-regulated and 22 down-regulated (Table S2). A more detailed representation of the up and down regulation of miRNAs is presented in Table 2.

An interesting observation is that three of the four up-regulated miRNAs (miR-210, LET-7a, miR-181b) (Table 3) were also the most significant miRNAs, as shown in Table 2. The raw data of global miRNAs expression analysis is available at the Gene Expression Omnibus [15].

**Figure 3:**
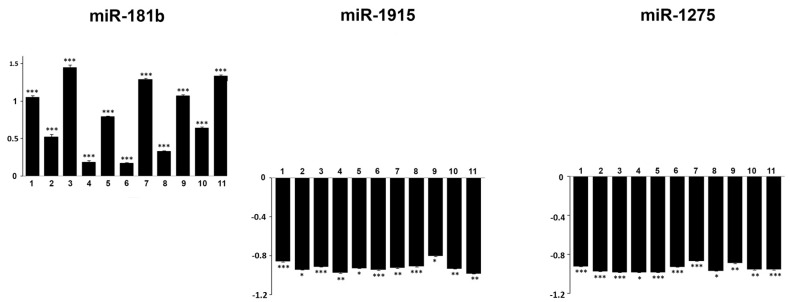
The expression levels of miR-181b, miR-1915 and miR-1275 in Ewing’s Sarcoma samples and in normal MSCs assessed by quantitative RT-PCR. The examined miRNAs were highly deregulated in ES primary tumors (* *p* < 0.05; ** *p* < 0.01; *** *p* < 0.001).

Quantitative RT-PCR (qRT-PCR) was performed using the same total of RNA employed for the microarray analysis. Due to the limited amount of residual available RNA, only 11 ES samples were tested in duplicate qRT-PCR reactions for the expression of miR-181b, miR-1915 and miR-1275 (Figure 3).

These three aberrantly regulated miRNAs were validated using the qRT-PCR as a different molecular method because they appeared to be significant in our experimental results (Table 2 and Table 3).

Overall, the qRT-PCR data showed a similar trend in miRNAs expression as the one revealed by microarray analysis. Taken together these results show a modulated, deregulated expression of a series of miRNAs clusters playing important roles in ES. In particular, a verified group of miRNAs were up-regulated.

### 2.3. Target Gene Prediction of Deregulated miRNAs in ES

Afterwards, we analyzed the presence of the target prediction genes associated with the miRNAs which were deregulated in the selected tissue samples originated from ES patients. The research for differentially regulated miRNAs-target gene interaction was performed for each miRNA that was strongly, experimentally validated in the previous analysis, using the miRTarbase prediction tool [12]. We identified numerous target genes showing a significant match in the database (Table 4) that were assigned to 126 more evident target genes belonging to the Kyoto Encyclopedia of Genes and Genomes (KEGG) pathways (Table S3).

**Figure 4:**
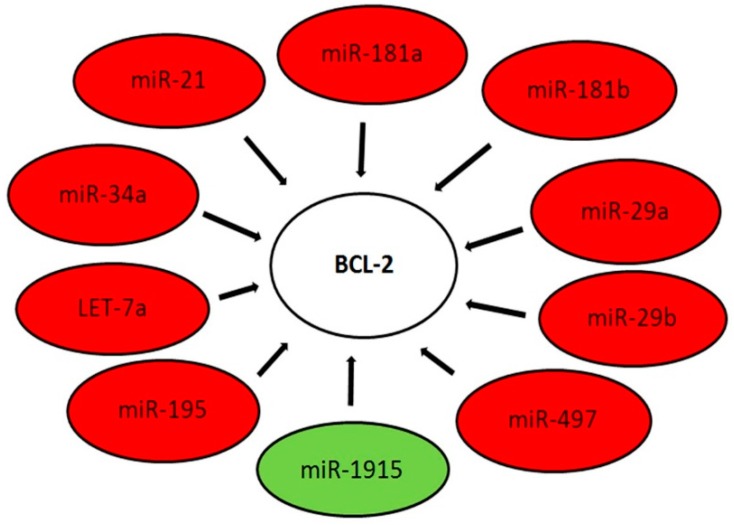
Schematic representation of the 10 deregulated miRNAs and the predicted *BCL-2* target gene as suggested in this work using miRNAs expression profile data, miRTarBase and a thorough examination of the literature. The *BCL-2* gene regulatory interaction network formed by the nine significantly up-regulated miRNAs (red) and one significantly down-regulated miRNA (green).

Some of the analyzed miRNAs such as miR-1286, miR-1275, miR-665, miR-602 and miR-1248 did not show any corresponding target gene (Table S4). Some miRNAs such as miR-937, miR-1303, miR-1908, miR-1915, miR-762 and miR-379 had only been experimentally validated in previous studies [12] by next-generation sequencing experiments (NGS), while other miRNAs were validated by alternative methods (Table S4). All these miRNAs had not been validated by powerful confirmatory methods such as “reporter assay”, “western blot”, “qRT-PCR” or “microarray analysis”. It was shown that these differentially expressed gene targets have a wide range of functions. In addition, we observed that among the validated miRNAs-target interactions with weaker supporting evidence, 10 target genes had previously been shown to be associated with, or involved in ES (Table S4). We would underline that in some cases, more than one miRNA acts on the same target gene but in different ways. To note, our study suggests that certain aberrantly expressed miRNAs all target the *BCL-2* family genes in ES patients. As shown in Figure 4, we identified the *BCL-2* gene as being a specific target of miR-21, miR-181a, miR-181b, miR-29a, miR-29b, miR-497, miR-195, let-7a, miR-34a and miR-1915 (Figure 4 and Table 4).

## 3. Discussion

In the current study we sought to analyze the global miRNAs expression profiles in 20 tumor tissue samples from ES patients and compared these profiles to human MSCs samples, used as controls. Using the microarray approach we identified 58 differentially expressed miRNAs, including miR-21, miR-30b, miR-27a, miR-106b, miR-181a/b, miR-130a, let-7e, let-7b, let-7f, let-7g, let-7a and miR-34a, all of them up-regulated. Many of them had already been identified by other studies and their possible role in the development of ES tumors had already been taken into consideration, such as for miR-21 and the various members of the let-7 family [16]. We should underline that amongst the identified miRNAs, some exhibit discordant expression patterns if compared to those reported in other studies, such as miR-21 [17]. We may speculate that the differences in expression obtained in the two studies are ascribable to the diversity of samples and protocols used. We must also point out that our samples come from biopsies of patients in the early stage of the disease who have not yet been treated. Another very relevant issue is that our study involved the use of formalin-fixed, paraffin-embedded (FFPE) Ewing Sarcoma tissue. A recent study demonstrated that miRNAs are very well conserved in these tissues and that this type of sample is a useful tool to study tumors at various stages [18]. Usually the availability of Ewing Sarcoma samples to support biomedical research is a very challenging issue due to tumor rarity. Although frozen tissues are preferred over paraffin-embedded ones for molecular investigations (due to the possible nucleic acid degradation related to fixation, paraffin-embedding and decalcification processes), FFPE tissue samples are easier to deal with as encouraging results from Gomes *et al.* [19] seem to indicate, a good correlation between FFPE and frozen samples is plausible. Moreover, some other studies confirmed that the tissue storage times (2–9 years) did not seem to affect the number of detected microRNAs in the FFPE samples compared to matched frozen samples [19,20,21]. It must be also be emphasized that it was possible to obtain samples from patients who were not receiving any therapy or showing metastases and/or possible consequent resistance from drug.

Recent studies have shown that miR-21 is over-expressed in several types of cancer and contributes to tumor resistance in chemotherapy. Up-regulated miR-21 levels are accompanied by marked reductions of PTEN and/or PDCD4 expression (both regulated by miR-21) [22]. In a different study, depletion or down-regulation of miR-21 by a specific antisense oligonucleotide, has been demonstrated to result in decreased cell proliferation, inhibited cell-cycle progression and increased cell apoptosis [23]. More interestingly, miR-21 functions as an oncogene and modulates tumorigenesis through the regulation of the *BCL-2* gene. In particular, *BCL-2* up-regulation may be caused by miR-21 over-expression, so preventing the tumor-cell apoptosis that would otherwise be induced by chemotherapy drugs [24]. Aberrantly regulated miR-181a and miR-181b have been correlated with cancer progression and poor survival in cervical cancer, ovarian cancer and breast cancer. The function of miR-181a and miR-181b are complex, displaying either pro-proliferative or pro-apoptotic roles under specific physiological conditions and in different types of cancers. Many reports demonstrated that miR-181a and miR-181b exhibit their action via targeting several genes such as BCL-2 and *MCL-1* by direct binding to their 3′-UTR [25]. Furthermore, the over-expression of miR-181a/b are partly responsible for increased drug resistance preventing apoptosis by targeting the same *BCL-2* gene [25]. Recently, it has been shown that CD99 counteracts EWS-FLI-1 in controlling NF-κB signaling through the miR-34a [26]. Marino et al. [27] have shown that miR-34a is associated with cyclin D1 and ki-67 expression; in particular they demonstrated that the expression of miR-34a was lower in metastases than in primary tumors and that this phenomenon was inversely correlated with the expression of cyclin D1 and Ki-67. It is well known that the main role of miR-34a is the control of cellular proliferation. Furthermore, miR-34a seems to be involved in controlling cell apoptosis via targeting *BCL-2*. Ingenuity Pathway Analysis (IPA) of miR-34a, miR-181a and miR-146a network shows that these miRNAs are closely linked to each other, to *BCL-2* and to mitochondria, because the BCL-2 family members are involved in maintaining mitochondrial integrity [27,28]. Qiu *et al.* [29] showed that miR-29a and miR-29b act via multi-target genes related to the extracellular matrix such as *COL4A1*, *COL3A1* and *SPARC* suggesting their possible role in migration, invasion and tumor metastasis. More recently, it has been shown that miR-29a/b targets the 3’-untranslated region of the anti-apoptotic BCL-2 family protein [30]. In a recent report it was shown that Let-7 plays various roles in the regulation of the cellular apoptosis through targeting the anti-apoptotic protein BCL-2 in many cell types [31]. Importantly, the Let-7 family is involved in the maintenance and/or differentiation of cancer stem cells (CSCs) and it was suggested that these genes are probably involved in chemoresistance of CSCs [32,33]. MiR-195 is up-regulated in different types of cancer (metastatic melanoma, gastric cancer, prostate cancer, lung cancer, colorectal cancer and hepatocellular carcinoma) and this result is in agreement with our findings [34]. MiR-195 functions as a tumor suppressor miRNA by targeting several genes involved in cell cycle acceleration and anti-apoptotic factors including *BCL-2*. Another *BCL-2* targeting miRNA is miR-497 demonstrated to directly hybridize to the predicted 3′-UTR target sites of this gene. Taking all this data into account, it is possible to assume that all these different up-regulated miRNAs can act together targeting the same gene, *BCL-2*. It is very well-known that BCL-2 family proteins can either suppress or promote apoptosis. More recently, many reports support the evidence that BCL-2 acts to regulate cancer cell invasion and metastasis through mitochondrial metabolism [35]. It is also relevant to note that the BCL family contribute to anoikis evasion. Anoikis resistance is considered to be a critical step in ES tumor progression [36]. For all these reasons we suggest that *BCL-2* might become a “new” predicted target gene for ES. 

Interesting highlights in our findings are that let-7A/E and miR-181b are up-regulated in >90% of ES patients. All these observations imply further research to experimentally validate the described data. Furthermore, miR-21 and miR-29a regulate several genes associated with ES, such as the IGF1 pathway genes, *FLI-1*, *EWSR1* and the *EWS-FLI-1* fusion genes [18]. Preclinical animal studies already have suggested that let-7a can be a potential candidate for miRNA-based therapies [5,17]. Interestingly, many of the deregulated miRNAs reported in these studies are located in chromosomal regions already described as involved in ES-specific translocations (Table S5). The expression pattern of certain miRNAs, such as miR-34c and let-7b, was presumed to be closely associated to the chromosomal regions involved in the ES-specific translocations. All these findings are concordant with the data provided by the present study. Although this finding does not imply that genes for miRNAs clusters are associated with genomic alterations, those located in the regions involved in chromosomal translocations might be aberrantly expressed in translocation-caused Ewing sarcoma. This effect could be due to changes in neighboring regulatory sequences or to gene-transcription factors [34,36]. These miRNAs may therefore be related to the general mechanisms of tumor development and carcinogenesis, since many of the deregulated miRNAs have repeatedly been identified in a variety of malignancies other than ES (Mitelman Database of Chromosome Aberrations in Cancer. [37]. We should underline that some of these miRNAs (Table 3) are expressed in the 90% of the ES patients, such as the up-regulated miR-210 (11p15.5), Let-7a (9q22.32), Let-7e (19q13.41), miR-181b (1q32.1) and the down-regulated miR-1908 (11), miR-659 (22q13.1) and miR-937 (8q24.3). MiR-210 is associated with tumor hypoxia and correlated with many tumors [38]. Sun Y *et al.* [39] demonstrated that HIF-1α and miR-210 showed a significant increase under hypoxic condition. They observed that the inhibition of HIF-1α decreased the miR-210 expression and autophagy and that the silencing of miR-210 up-regulated *BCL-2* expression. MiRNA-1908 functions as an oncogene in several tumors by repressing the PTEN pathway [40]. Moreover, some authors identified miR-1908, miR-199a-5p and miR-199a-3p as endogenous promoters of metastatic invasion, angiogenesis and colonization in melanoma [41]. There are no reports regarding miR-659 and its association with cancer, while it was shown to be correlated with neurodegenerative disorders [42]. Recently, it has been demonstrated that miR-937 is highly expressed in MSCs [43]. In our study, the expression profiles of 3 miRNAs such as miR-181b, miR-1915 and miR-1275 were confirmed by qRT-PCR. We have already described miR-181b and its target multiple apoptosis genes, such as *BCL-2* and *MCL-1.* This miRNA was also associated to chronic lymphocytic leukemia and was shown to promote chemoresistance in pancreatic ductal adenocarcinoma cells and breast cancer [24]. MiR-1915 targets *BCL-2* and modulates multidrug resistance of human colorectal carcinoma cells [44]. Further studies suggested a negative regulation of *BCL-2* by p53 via-miR-1915 to induce apoptosis. In the present study, miR-1915 was down-regulated in both its immature and mature form and miR-1915 was significantly deregulated. Very recently, Fawzy *et al.* [45] showed that *IGF1R* is a direct target of miR-1275. They suggested that miR-1275 can control hepatocellular carcinoma tumor growth partially through regulating the oncogene *IGF2BPs* and *IGF1R*. It is well known that the IGF1R pathway is deregulated in ES and several studies are evaluating it as a potential target for therapy. Consequently, miR-1275 could play a role in ES tumor progression by regulating *IGF1R* [46]. Katsushima *et al.* [47] recently demonstrated that miR-1275 was downregulated during Glioma stem-like cell differentiation, together with the upregulation of its target, *CLDN11*, via PRC2-H3K27me3. Several studies have highlighted the essential contribution of PRC2-H3K27me3 to the repression of developmental regulator genes that enable successful cell fate reassignment [48,49]. MiR-1275 could be postulated to play some critical role in ensuring highly-selective regulation of one or more target genes and perhaps determining heterogeneous cell fate.

In conclusion, our study provides new information on miRNAs expression and has demonstrated that 58 differentially expressed miRNAs were found in the primary tumor tissue of ES patients when compared to MSCs, suggesting that these molecules may potentially serve as candidate tumor biomarkers in ES and/or as therapeutic targets. 10 miRNAs were present in most of our patients (four miRNAs were up-regulated and six down-regulated). Although several groups have already identified and characterized deregulated miRNAs expression in ES using different approaches, a limiting factor has been the scarce availability of patient tissue’s samples for research given the low incidence of the tumor (approximately one case per million in the general population).

In this study, the microarray analysis was performed on 20 ES primary untreated tumors. Subsequent miRTarbase analysis suggested a number of predicted target genes that could be critical in ES pathogenesis and future treatment. Our investigation into miRNAs and the miRNAs interaction network has revealed the co-regulation of subpathways by certain, corresponding up-regulated and down-regulated miRNAs. Further functional investigations of miRNAs and multiple miRNAs target pathways are needed to achieve a wider knowledge of their responsibility in the complex interaction processes in disease-related regulatory pathways [50].

The unique miRNAs expression patterns identified, including the over-expressed miRNAs clusters in ES and their predicted target genes, warrant further investigation to develop a better understanding of the oncogenic mechanism and to inspire future therapeutic strategies for ES.

## 4. Experimental Section

### 4.1. Patients

The eligibility criteria of the patients enrolled in this study were as follows: diagnosis of ES according to the World Health Organization (WHO) classification; age younger than 40 years; no starting therapy; no evidence of metastasis. This retrospective study included eligible patients who were diagnosed at the Department of Pathology of the Orthopedic Institute Gaetano Pini, Milan, Italy, from April 1995 to April 2011, with complete clinical-pathological and histological data (Table 1). This series included 20 patients (6 women and 14 men, mean age ± standard deviation 23.15 ± 10.757 years), from which a tissue sample of primary tumor was obtained. Only adequate biopsies at the time of diagnosis before any treatment were selected for the study.

Paraffin-tumor tissue samples, without information linked to the patients’ identities, except for tumor diagnoses, histological and molecular genetic data were used. All tumor tissue samples were fixed in 10% buffered formalin and paraffin-embedded (FFPE) and 4 micron sections were cut for haematoxylin-eosin (H & E) and immunohistochemical staining. Immunohistochemistry was performed by a BenchMark ULTRA automated slide stainer (Ventana, Tucson, AZ, USA) using ultraView Universal DAB Detection Kit (Ventana, USA) and the following primary antibodies: CD99 (mouse monoclonal, clone HO36-1.1, Leica, Milton Keynes, MK 14-6FG, UK), HBA71 (Mic-2; mouse monoclonal, clone 12E7, Dako, Glostrup, Denmark), FLI-1 (mouse monoclonal, MRQ-1, Cell Marque, Rocklin, CA, USA), caveolin (rabbit monoclonal, SP43, Spring Bioscience, Atlanta, GA, USA), CD45 (LCA, 2 mouse monoclonal cocktail, clones 2B11 and PD7/26, Ventana, USA), MyF4 (NCL-L-MYF4, mouse monoclonal, clone LO26, Leica), desmin (mouse monoclonal, DE-R-11, Ventana), NSE (mouse monoclonal, clone E27, Ventana), pan-cytokeratin (mouse, antibody cocktail, clones AE1/AE3 and PCK26, Ventana). The cases of tumor type-specific fusion genes (*EWS/FLI-1)* were also detected by reverse transcription-polymerase chain reaction (RT-PCR). In the study, human MSC commercial lines from four different donors were included and used for comparison purposes. The study was approved by the institutional review board of The Gaetano Pini Hospital (Milano, Italy) (ID. Number 3117-26 May 2010). All clinical investigations were conducted according to the principles expressed in the Helsinki declaration. Written informed consent was obtained from children’s parents, patients and controls.

### 4.2. Mesenchymal Stromal Cell (MSC) Culture

Human mesenchymal stem cells (MSC) lines were obtained from Lonza and American Type Culture Collection (ATCC) (Manassas, VA, USA). Normal MSC were isolated from normal (non-diabetic) adult human bone marrow withdrawn from bilateral punctures of the posterior iliac crests of normal volunteers. We used the MSCs between passages 2–4. MSCs were cultured in proprietary media in according to the recommendations of Lonza and ATCC. MSCs purity was determined by flow cytometry and chondrogenic, osteogenic, adipogenic differentiation capabilities.

### 4.3. RNA Extraction and miRXplore™ Microarrays

Total RNA extraction was performed using the miRNeasy kit (Qiagen, Valencia, CA, USA) for MSCs according to the manufacturer’s instructions. The RNAs were isolated from human FFPE tissue samples with the use of the miRNeasy^®^ FFPE Kit (Qiagen) following the guidelines. RNA quality and integrity were determined using the Agilent 2100 Bioanalyzer (Agilent Technologies, Santa Clara, CA, USA) and quantified with the Qubit^®^ flurometer (Life Technologies, Carlsbad, CA, USA). Only samples which had an RNA integrity number (RIN) score greater than 9.5 [51] were used for the study. Moreover, according to miRXplore Microarray method (Milteny Biotec, Bergisch Gladbach, Germany), all samples were labelled, hybridized and then run in duplicate on an Agilent V3 miRNA array. The labelling of RNA/miRNA was performed using the miRCury Power Labelling Kit (Exiqon, Vedbaek, Denmark) according to the manufacturer`s instructions. The miRXplore™ Universal Reference (Miltenyi Biotec, Bergisch Gladbach, Germany) was labelled with Hy3 and experimental samples were labelled with Hy5. The miRXplore™ Universal Reference represents a defined pool of 954 synthetic microRNAs for comparison of multiple samples. The total labelled RNA mix (Universal Reference as control and the sample of interest) was hybridized in a dual-colour approach to miRXplore microarrays (Table S6). Hybridization was performed using an automated hybridization instrument following the manufacturer’s instructions (a-Hyb™ Hybridization Station, Miltenyi Biotec). Briefly, microarray processing in the a-Hyb was performed as follows: incubation in Pre-Hyb Solution (Miltenyi Biotec) for 5 min at 42 °C, hybridization with the labelled RNAs for 960 min at 42 °C, washing with Wash Buffer I (Miltenyi Biotec) for 1 min at 10 °C (2 cycles) and with Wash Buffer II (Miltenyi Biotec) for 1 min at 10 °C (2 cycles). The pump speed for all incubations was set to 1 mL/min.

### 4.4. Microarray Analysis

Fluorescence signals of the hybridized miRXplore™ microarrays were detected using Agilent’s Microarray Scanner System (Agilent Technologies). Signal quantification of hybridized miRXplore™ microarrays was done with the ImaGene software Version 9.0 (BioDiscovery, Los Angeles, CA, USA) and mean signal and mean local background intensities were obtained for each spot. Low-quality spots were flagged and excluded from data analysis. Unflagged spots were further analysed with the miRXplorer^®^ software (Miltenyi Biotech Microarray Service, Colonia). The analysis includes background correction, data normalization, calculation of the Hy5/Hy3 ratios as well as re-ratio calculation. As an additional quality filtering step, only spots/genes having a signal equal to or higher than the 67% of the background signal intensities have been taken into account for the calculation of the Hy5/Hy3 ratio (Tables S1 and S2). The microarray data has been submitted to GEO [15].

### 4.5. Quantitative Real-Time PCR

For miRNA analysis 10ng total RNA was used for complementary DNA preparation with a TaqMan MicroRNA reverse transcription kit and a miRNA-specific primer (Table S7).

Quantitative real-time polymerase chain reactions (qRT-PCRs) were performed for each sample using the TaqMan^®^ MicroRNA assays and TaqMan MicroRNA RT kit (Applied Biosystems, Foster City, CA, USA), according to the manufacturer’s protocol in the IFOM-IEO Campus (Milan, Italy). Briefly, the thermal cycler program for reverse transcription was set at 16 °C for 30 min, 42 °C for 30 min and 85 °C for 5 min followed by a 4 °C hold. The amplification protocol was 95 °C for 10 min followed by 40 cycles of 95 °C for 15 s followed by annealing/extension at 60 °C for 60 s. Amplification data was then analyzed in order to determine the detection threshold cycle (*C*_t_) for each sample. Relative expression levels were calculated according to the comparative threshold cycle (*C*_t_) method using the ubiquitous small nucleolar RNA U6, which is considered to be an appropriate endogenous reference control in ES. The mean optical background level for each array was subtracted from the signal intensity of the reference control. Δ*C*_t_ = *C*_t_(miRNA) − *C*_t_(U6). Moreover, the respective ΔΔ*C*_t_ (ΔΔ*C*_t_ = mean Δ*C*_t_ patient’s group-mean Δ*C*_t_ control group) [50] was calculated for the patients’ group and for the primary MSC cultures used as control group. Finally, fold change (FC) expression of each miRNA (2 ΔΔ*C*_t_) was determined. FC ≤ −0.5 and FC ≥ 1.2 were considered differentially expressed between the patients’ group and primary MSC cultures. All the experiments were assayed in triplicate.

### 4.6. Target Gene Prediction of Deregulated miRNAs in ES

The miRTarBase database [52], a resource for information of experimentally-validated miRNA target interaction was used to analyze the target gene of the deregulated miRNAs [14].

The genes predicted by strong evidence using Western Blot, Reporter assay and qRT-PCR were selected as deregulated miRNA targets in ES.

### 4.7. Statistical Analysis

Exact Mann-Whitney *U* test was used to identify the miRNAs that were expressed differently in the two groups (ES samples and MSCs controls) and *p*-value ≤0.05 was considered to indicate a statistically-significant difference. The *p*-values of the selected miRNAs were corrected for controlling the false discovery rate (FDR) by using the Simes multiple-test procedure [16].

## 5. Conclusions

Main text. Using paraffin-embedded tissues from 20 ES patients, this study has identified several potential target miRNAs and a gene *BCL-2* that might be considered a novel critical biomarker for ES pathogenesis. Further functional investigations are required to clearly define the role of miRNAs and multiple miRNAs target pathways and the implication in the complex interaction processes in disease-related regulatory pathways.

## Figures and Tables

**Table 1:** Clinical, morphological, immunohistochemical and molecular characteristics of ES patients.

Patient	Age	Gender	Primary Site	HBA71	CD99	CD45	VIM	NSE	NF	CAM 5.2	Myogenin	FLI-1	Pattern
1	16	M	Femur	+	+	−	+	+	−	−	−	+	Diffuse
2	40	M	Foot	+	+	−	+	+/−	−	−	−	+	Diffuse, filigree
3	28	F	Bone pelvis	+	+	−	+	−	−	−	−	+	Diffuse, filigree
4	19	M	Bone pelvis	+	+	−	+	+	+	−	−	+	Diffuse
5	15	M	Arm	+	+	−	+	−	−	−	−	+	Diffuse, pseudorosettes
6	40	M	Arm	+	+	−	+	+	+	−	−	+	Diffuse, filigree
7	14	M	Humerus	+	+	−	+	+/−	−	−	−	+	Diffuse
8	24	M	Scapula	+	+	−	+	+/−	+/−	−	−	+	Diffuse, pseudorosettes
9	30	M	Humerus	+	+	−	+	+	+	+	−	+	Large cells
10	30	M	Bone pelvis	+	+	−	+	+	−	−	−	+	Diffuse, filigree
11	38	M	Femur	+	+	−	+	−	−	−	−	+	Diffuse, large cells
12	12	F	Bone pelvis	+	+	−	+	+	−	−	−	+	Diffuse
13	26	M	Femur	+	+	−	+	+	−	−	−	+	Diffuse, filigree
14	18	M	Rib	+	+	−	+	+	−	−	−	+	Diffuse
15	5	F	Bone pelvis	+	+	−	+	+	−	−	−	+	Filigree
16	15	F	Sacrum	+	+	−	+	+/−	−	−	−	+	Diffuse, pseudorosettes
17	9	M	Humerus	+	+	−	+	−	−	−	−	+	Diffuse
18	30	F	Bone pelvis	+	+	−	+	−	+/−	−	−	+	Diffuse
19	38	M	Tibia	+	+	−	+	−	−	−	−	±	Diffuse
20	16	F	Iliac wing	+	+	−	+	+/−	−	−	−	+	Diffuse, pseudorosettes

+ >30%; +/− 10%–30%; −/+ <10%; − negative. HBA71: monoclonal antibody that recognizes a cell-surface glycoprotein, p30/32MIC2; CD99: transmembrane glycoprotein; CD45: Leucocyte Common Antigen; Vim: Vimentine; NSE: Neurone Specific Enolase; NF: Neurofilaments; CAM 5.2: Keratin 8-18.

**Table 2:** Differentially expressed miRNAs in Ewing’s Sarcoma compared to control mesenchymal stromal cells.

miRNA	Chromosomal Locus	Mean	Fold Change (Log_2_ *vs.* ES/cont)	*p* Value (*vs.* ES/cont)	up/down	miRNA	Chromosomal Locus	Mean	Fold Change (Log_2_ *vs.* ES/cont)	*p* Value (*vs.* ES/cont)	up/down
LET-7b	22q13.31	1.14224	1.70097757	0.0453604	up	miR-222	Xp11.3	1.1226405	2.160497442	0.0453604	up
miR-130a	11q12.1	0.145273	1.802940185	0.0227743	up	miIR-29a	7q32.3	0.7785172	3.211108837	0.0071523	up
miR-181a	1q32.1	0.40997	3.483396018	0.0227743	up	miR-30e	1p34.2	0.1530775	1.284191047	0.0176925	up
miR-195	17p13.1	0.2839247	2.364840938	0.0453604	up	miR-34a	1p36.22	0.0985494	2.602895388	0.0291737	up
miR-21	17q23.1	2.1959104	2.162445617	0.0133634	up	miR-376c	14q32.31	0.1997549	1.391755139	0.0099755	up
miR-210	11p15.5	0.0444813	2.037000568	0.0071523	up	miR-1248	3q27.3	11.1565844	2.026344903	0.0291737	up
miR-23a	19p13.13	0.5450406	1.557042028	0.0291737	up	miR-320d	13q14.11	0.3488692	1.298152279	0.0099755	up
miR-27a	19p13.13	0.9184833	1.646126413	0.0227743	up	miR-330-3p	19q13.32	0.0118817	−0.676200792	0.0291737	down
miR-27b	9q22.32	0.2785094	1.701162712	0.0291737	up	miR-572	4p15.33	0.1956862	−0.888176812	0.0227743	down
miR-30b	8q24.22	2.401562	2.11959445	0.0291737	up	miR-602	9q34.3	0.1087883	−0.836221025	0.0291737	down
miR-30c	6q13	0.1031819	1.54622356	0.0453604	up	miR-638	19p13.2	8.5804901	−1.077583316	0.0099755	down
miR-361-5p	Xq21.2	0.0385326	1.221056398	0.0227743	up	miR-659	22q13.1	0.1805629	−1.596659699	0.0365142	down
LET-7a	9q22.32	1.8205888	2.259943742	0.0099755	up	miR-663	20p11.1	13.9526609	−1.339518478	0.0050819	down
LET-7f	9q22.32	0.9668449	1.578072257	0.0133634	up	miR-183	7q32.2	0.0463532	−1.263442073	0.0453604	down
miR-146b-5p	10q24.32	0.045396	1.619529112	0.0133634	up	miR-665	14q32.2	0.6595578	−1.077518251	0.0133634	down
miR-19b	13q31.3	0.3502126	1.506461715	0.0365142	up	miR-212	17p13.3	0.0648259	−1.386056739	0.0071523	down
miR-106b	7q22.1	0.1227391	1.374387113	0.0227743	up	miR-223	Xq12	0.4399229	−1.610242879	0.0133634	down
miR-199a-5p	19p13.2	0.2432319	2.361398646	0.0291737	up	miR-675	11p15.5	0.1259554	−1.219277168	0.0176925	down
miR-379	14q32.31	0.0165888	1.446876011	0.0227743	up	miR-34c-3p	11q23.1	2.7175848	−1.780710627	0.0071523	down
miR-497	17p13.1	0.0887834	2.468076256	0.0227743	up	miR-937	8q24.3	1.2879218	−2.295916752	0.0453604	down
miR-29b	7q32.3	1.6144369	2.937173211	0.0071523	up	miR-18b *	Xq26.2	0.620149	−1.110742814	0.0453604	down
miR-151-5p	8	0.0494687	1.690015675	0.0099755	up	miR-1228 *	12	70.6243849	−1.25250658	0.0227743	down
miR-301a	17q22	0.0222602	1.260984605	0.0291737	up	miR-1275	6	234.2215799	−1.668322452	0.0291737	down
LET-7e	19q13.41	0.1148639	1.281646088	0.0291737	up	miR-1286	22	7.7291593	−1.495022244	0.0291737	down
LET-7g	3p21.1	0.4386578	1.627573169	0.0176925	up	miR-1303	5	4.2065158	−1.319485649	0.0176925	down
miIR-128	2q21.3	0.10438	1.904723965	0.0365142	up	miR-1908	11	328.65951	−1.712608661	0.0133634	down
miR-181b	1q32.1	0.1493547	2.87116484	0.0071523	up	miR-1915 *	10p12.31	2.3107252	−1.26347519	0.0453604	down
miR-196a	17q21.32	0.0566933	1.723288209	0.0050819	up	miR-1915	10p12.31	28.386301	−1.609043615	0.0099755	down
miR-199b-3p	19p13.2	0.7680752	2.607320218	0.0176925	up	miR-762	16	15.9034606	−0.965705224	0.0133634	down

ES: Ewing’s Sarcoma; cont: control mesenchymal stromal cells; * identifies mature form of miRNAs.

**Table 3:** miRNAs deregulated in >90% of Ewing’s Sarcoma (ES) patients.

miRNA	up/down
miR-210	up
LET-7a	up
LET-7e	up
miR-181b	up
miR-659	down
miR-665	down
miR-937	down
miR-1275	down
miR-1915	down
miR-1908	down

**Table 4:** Target Gene Prediction of Deregulated miRNAs.

miRNA	Target Gene		miRNA	Target Gene	
	a	b		a	b
let-7B	*CDC34*, *CDC25A*, *CCND1*	*IGF2BP1*, *HMGA2*, *CDK6*, *BCL7A*, *NR2E1,* *PRDM1*, *HRAS*, *CYP2J2*	miR-128	*BMI1*, *TGFBR1*, *FBXW7*	*DCX*, *RELN*, *WEE1*, *KLF4*, *E2F3*, *EGFR*
let-7A	*EWSR1*, *NF2*, *KRAS*, *E2F2*, *IL6*, *CCR7*, *BCL2*, *HMGA2-A1*	*MYC*, *NKIRAS2*, *ITGB3*, *NRAS*, *PRDM1*, *UHRF2*, *DICER1*	miR-181b	*BCL2*, *TCL1A*, *RNF2*	*E2F1*, *PLAG1*, *KAT2B*, *TIMP3*, *MAP3K10*, *TMED7*
let-7F		*PRDM1*, *KLK6*, *KLK10*	miR-196A	*HMGA1*, *HMGA2*, *HOXC8*, *CDKN1B*	*HOXA5*, *HMOX1*, *BACH1*, *HOXB7*
					*HOXA7*, *HOXB8*, *ANXA1*
let-7E		*CCND1*, *HMGA2*, *WNT1*	miR-199A-5p	*IKBKB*, *HIF1A*, *CAV1*, *ERBB2*, *GSK3B*, *JAG1*	*DDR1*, *MAP3K11*, *SIRT1*, *SMARCA2*, *KL*, *HSPA5*
let-7G	*MYC*, *HMGA2*, *CDKN2A*	*IGF2BP1*, *GAB2*, *FN1*, *BMI1*	miR-199A-3p	*CD44*, *MET*, *MTOR*	*SMARCA2*, *FUT4*, *CAV2*, *MAPK1*
	*BCL2L1*, *COL1A2*				*MAPK8*, *MAPK14*
miR-130a	*HOXA5*, *RUNX3*, *PPARG*	*ATXN1*, *MEOX2*, *HOXA10*, *CSF1*, *KLF4*, *SMAD4*	miR-222	*CDKN1B*, *SOD2*, *MMP1*, *CDKN1C*, *KIT*, *TMED7*, *TIMP3*, *PTEN*	*STAT5A*, *FOXO3*, *FOS*, *ESR1*, *BBC3*, *DIRAS3*, *ETS1*, *CERS2*, *TRPS1*
miR-181A	*BCL2*, *CDKN1B*, *RNF2*, *RALA*	*PLAG1*, *PROX1*, *ZNF763*, *BCL2L11*, *HRAS*, *KLF6*	miR-29A	*MCL1*, *BCL2*, *PPM1D*, *CDK6*, *DNMT3A-3B*, *COL4A2-A1*, *SPARC*, *PIK3R1*, *SERPINB9*	*PTEN*, *BACE*, *CD276*, *SFRP2*, *DKK1*, *GLUL*, *LPL*, *KREMEN2*, *ADAMTS9*, *ITAGA11*, *MYCN*, *SAPCD2*
miR-195	*BCL2*, *WEE1*, *E2F3*, *CDK6*, *RUNX2*, *RAF1*	*CCND1*, *CCL4*, *SLC2A3*, *TBCCD1*, *CCND3*, *BCL2L2*	miR-30E	*MYBL2*, *NOTCH1*	*UBE2I*, *SNAI1*, *MUC17*, *TP53*
miR-21	*BCL2*, *SOX5*, *E2F1*, *PTEN*, *TGFBR2*, *TIMP3*, *PDCD4*	*CDC25A*, *RASGRP1*, *RPS7*, *MTAP*, *RECK*, *APAF1*, *TPM1*, *ANKRD46*, *BTG2*, *BMPR2*, *CDK2AP1*, *DAXX*, *EIF4A2*, *ISCU*, *JAG1*, *LRRFIP1*, *MSH2*, *MSH6*, *NFIB*, *PPARA*, *RHOB*, *SERPINB5*, *SMARCA4*, *SPRY2*, *TGFB1*, *TOPORS*, *TP63*, *TPM1*	miR-34A	*MYC*, *BCL2*, *NOTCH1*, *JAG1*, *MET*, *CDK4*, *CDK6*, *CCND1*, *E2F3*, *NOTCH2*, *PDGFRA*, *MAP3K9*	*MYB*, *CCNE2*, *WNT1*, *SIRT1*, *PEA15*, *HNF4A*, *MAGEA3*, *MAGEA2*, *MAP2K1*, *MYCN*
miR-210	*FGFRL1*, *BDNF*, *PTPN1*, *ISCU*, *E2F3*	*RAD52*, *NPTX1*, *MNT*, *EFNA3*, *VMP1*, *P4HB*, *NCAM1*, *GPD1L*, *CPEB2*, *DDAH1*	miR-376C	*IGF1R*, *ACVR1C*, *TGFBR1*, *GRB2*	*TGFA*
miR-23A	*POU4F2*, *IL6R*, *PTEN*, *MYH1*, *MYH2*, *MYH4*	*CELF1*, *HES1*, *FOXO3*, *FANCG*	miR-320D		*RBFOX2*, *GNAI1*
miR-27A	*FOXO1*, *PHB*, *SPRY2*, *IGF1*	*ZBTB10*, *MYT1*, *SP4*, *SP3*, *SP1*, *WEE1*, *FBXW7*, *THRB*	miR-330-3p	*CDC42*	*E2F1*, *CD44*, *VEGFA*, *NTRK3*
miR-27B	*ST14*, *CCNT1*, *MMP13*	*CYP1B1*, *PPARG*, *EDNRA*, *EYA4*, *PAX3*	miR-572	*CDKN1A*	
miR-30B	*BCL6*, *SOCS1*, *SNAI1*	*CAT*, *CCNE2*, *SMAD1*	miR-638	*OSCP1*, *SP2*, *SOX2*	
miR-30C	*UBE2I*, *SNAI1*	*SMAD1*, *HSPA4*, *TGIF2*, *HDAC4*	miR-659		*GRN*
miR-361-5p		*VEGFA*	miR-663	*TGFB1*, *JUNB*, *JUND*	*APC*, *PIK3CD*, *EEF1A2*, *MYL9*, *HRAS*
miR-146B-5p	*KIT*, *PDGFB*	*MMP16*, *TRAF6*, *IRAK1*	miR-183	*PDCD4*, *GSK3B*	*AKAP12*, *SRSF2*, *FOXO1*, *ITGB1*, *KIF2A*, *BTRC*
miR-19B	*PTEN*, *ATXN1*, *BMPR2*, *TLR2*	*ESR1*, *KAT2B*, *SOCS1*, *BCL2L11*, *TGFBR2*, *CUL5*	miR-212	*PTCH1*, *RB1*, *TJP1*, *MECP2*, *MYC*	*CCNB1*, *PEA15*, *CCNA2*, *ACHE*
miR-106B	*CDKN1A*, *E2F1*, *RB1*	*ITCH*, *APC*, *APP*, *KAT2B*	miR-223	*IGFR1*, *FOXO1*, *PARP1*, *NFIA*, *MEF2C*	*CHUK*, *STMN1*, *LMO2*, *E2F1*
	*TCEAL1*, *JAK1*, *BCL2L11*	*VEGFA*, *PTEN*, *CASP7*			*RHOB*, *FBXW7*, *ARTN*
miR-497	*RAF1*, *RUNX2*, *MAP2K1*, *BCL2*, *IGF1R*	*WEE1*, *EIF4E*	miR-675	*RUNX*, *CALN1*, *TGFBI*	*RB1*, *MITF*, *CDC6*
miR-29B	*COL1A1*, *BCL2*, *MCL1*, *SP1*, *TCL1A*, *CDK6*	*DNMT3B-3A*, *TET1*, *GRN*, *COL3A1*, *COL4A1*, *MMP2*, *ADAM12*, *NID1*, *HMGA2*, *BMP1*, *PTEN*, *PIK3CG*, *NKIRAS2*	miR-34c-3p		*CTNNB1*, *LEF1*, *AXIN2*
miR-151-5p	*ARHGDIA*	*MPL*, *N4BP1*, *E2F6*	miR-18b *		*ESR1*, *MDM2*, *SMAD2*, *FOXN1*
miR-301A	*NKRF*, *MEOX2*, *RUNX3*, *PTEN*	*SERPINE2*, *SMAD4*, *BCL2L11*	miR-1228 *		*MOAP1*
			miR-1915	*BCL-2*	*HIST2H3A*, *TMEM69*

* a = strong evidence (Western Blot, Reporter assay and qRT-PCR); b = less evidence (microarray, NGS, pSILAc, others).

## Supplementary Materials

Supplementary materials can be found at http://www.mdpi.com/1422-0067/17/5/656/s1. Table S1. Analysis of the change in expression of 954 miRNAs in 20 ES biopsies compared to MSCs commercial lines from 4 normal donors. (Miltenyi Biotec); Table S2. Analysis of the change in expression of 954 miRNAs in 20 ES biopsies compared to MSCs commercial lines from 4 normal donors, 58 miRNAs which are suitable for further evaluation (*p*-value ≤0,05 was considered statistically-significant) are highlighted in yellow; Table S3.Functional analysis [KEGG Pathway Enrichment] [Gene Ontology Enrichment]; Table S4. Target Gene prediction of deregulated miRNAs; Table S5. Summary of chromosomal loci of deregulated miRNAs in Ewing’s Sarcoma tissue; Table S6. miRXplore™ Microarrays: 954 microRNAs probe-sequences; Table S7. TaqMan^®^ MicroRNA Human Assays form Applied biosystem used in this study.
