# Supplementary material for: Ewing’s Sarcoma: An Analysis of miRNA Expression Profiles and Target Genes in Paraffin-Embedded Primary Tumor Tissue
Source: Int J Mol Sci. 2016 Apr 30;17(5):656. doi: 10.3390/ijms17050656 (PMC4881482; doi:10.3390/ijms17050656)
Supplement: Supplementary file 1 [file ijms-17-00656-s001.zip › ijms-126634-Supplementary Materials/ijms-126634-Supplementary Materials.pdf]

Supplementary Materials: Ewing's Sarcoma: An Analysis of miRNA Expression Profiles and Target Genes in Paraffin-Embedded Primary Tumor Tissue

Antonina Parafioriti, Caterina Bason, Elisabetta Armiraglio, Lucia Calciano, Primo Andrea Daolio, Martina Berardocco, Andrea Di Bernardo, Alessia Colosimo, Roberto Luksch and Anna C. Berardi

Table S1. Analysis of the change in expression of 954 miRNAs in 20 ES biopsies compared to MSCs from 4 normal donors. (Miltenyi Biotec).

Table S2. Analysis of the change in expression of 954 miRNAs in 20 ES biopsies compared to MSCs from 4 normal donors. 58 miRNAs which are suitable for further evaluation (p-value ≤ 0.05 was considered statistically-significant) are highlighted in yellow.

Table S3. Functional analysis [KEGG Pathway Enrichmennt] [Gene Ontology Enrichment].

Target Gene	Description	Target Gene	Description
CDC34	cell division cycle 34 homolog	*JAK1*	Janus kinase 1
CDC25A	cell division cycle 25 homolog A	*MAP2K1*	mitogen-activated protein kinase kinase 1
CCND1	Cyclin D1	*COL1A1*	Collagen. type I. alpha 1
EWSR1	Ewing sarcoma breakpoint region 1	*SP1*	Sp1 transcription factor
NF2	neurofibromin 2 (merlin)	*MCL1*	myeloid cell leukemia sequence 1 (BCL2-related)
KRAS	Kirsten rat sarcoma viral oncogene homolog	*TCL1A*	T-cell leukemia/lymphoma 1A
E2F2	E2F transcription factor 2	*ARHGDIA*	Rho GDP dissociation inhibitor (GDI) alpha
IL6	Interleukin 6 (Interferon. Beta 2)	*NKRF*	NFKB repressing factor
CCR7	chemokine (C-C motif) receptor 7	*MEOX2*	mesenchyme homeobox 2
BCL2	B-cell CLL/lymphoma 2	*RUNX3*	runt-related transcription factor 3
HMGA2	High mobility group AT-hook 2	*PTEN*	phosphatase and tensin homolog
HMGA1	High mobility group AT-hook 1.	*BMI1*	BMI1 polycomb ring finger oncogene
MYC	V-myc myelocytomatosis viral oncogene homolog (avian)	*TGFBR1*	transforming growth factor. beta receptor 1
CDKN2A	Cyclin-dependent kinase inhibitor 2A	*FBXW7*	F-box and WD repeat domain containing 7. E3 ubiquitin protein ligase
BCL2L1	B-cell CLL/lymphoma 2-LIKE PROTEIN 1	*TCL1A*	T-cell leukemia/lymphoma 1A
COL1A2	collagen. type I. alpha 2	*HOXC8*	homeobox C8
HOXA5	homeobox A5	*IKBKB*	inhibitor of kappa light polypeptide gene enhancer in B-cells. kinase beta
RUNX3	Runt related transcription factor 3	*HIF1A*	hypoxia inducible factor 1. alpha subunit (basic helix-loop-helix transcription factor)
PPARG	peroxisome proliferator-activated receptor gamma	*CAV1*	caveolin 1. caveolae protein. 22 kDa
CDKN1B	cyclin-dependent kinase inhibitor 1B (p27. Kip1)	*ERBB2*	v-erb-b2 erythroblastic leukemia viral oncogene homolog 2. neuro/glioblastoma derived oncogene homolog
RNF2	ring finger protein 2	*GSK3B*	glycogen synthase kinase 3 beta
RALA	v-ral simian leukemia viral oncogene homolog A (ras related)	*JAG1*	jagged 1
WEEE1	WEE1 homolog (S. pombe)	*MET*	met proto-oncogene (hepatocyte growth factor receptor)
E2F3	E2F transcription factor 3	*CD44*	CD44 molecule (Indian blood group)
CDK6	cyclin-dependent kinase 6	*MTOR*	mechanistic target of rapamycin (serine/threonine kinase)
RUNX2	Runt-Related Transcription Factor 2	*SOD2*	superoxide dismutase 2. mitochondrial
RAF1	v-raf-1 murine leukemia viral oncogene homolog 1	*MMP1*	matrix metallopeptidase 1 (interstitial collagenase)
SOX5	SRY (sex determining region Y)-box 5	*CDKN1C*	cyclin-dependent kinase inhibitor 1C (p57. Kip2)
E2F1	E2F transcription factor 1	*TMED7*	transmembrane emp24 protein transport domain containing 7
PTEN	phosphatase and tensin homolog	*DNMT3A*	DNA (cytosine-5-)-methyltransferase 3 alpha
TGFBR2	transforming growth factor. beta receptor II (70/80 kDa)	*DNMT3B*	DNA (cytosine-5-)-methyltransferase 3 beta
TIMP3	TIMP metallopeptidase inhibitor 3	*COL4A2*	collagen. type IV. alpha 2
PDCD4	programmed cell death 4 (neoplastic transformation inhibitor)	*COL4A1*	collagen. type IV. alpha 1
FGFRL1	fibroblast growth factor receptor-like 1	*PPM1D*	protein phosphatase. Mg2+/Mn2+ dependent. 1D
BDNF	brain-derived neurotrophic factor	*PIK3R1*	phosphoinositide-3-kinase. regulatory subunit 1 (alpha)
PTPN1	protein tyrosine phosphatase. non-receptor type 1	*SPARC*	secreted protein. acidic. cysteine-rich (osteonectin)
ISCU	iron-sulfur cluster scaffold homolog (*E. coli*)	*SERPINB9*	serpin peptidase inhibitor. clade B (ovalbumin). member 9

Table S3. *Cont.*

Target Gene	Description	Target Gene	Description
POU4F2	POU class 4 homeobox 2	*MYBL2*	v-myb myeloblastosis viral oncogene homolog (avian)-like 2
IL6R	interleukin 6 receptor	*NOTCH1*	Neurogenic locus notch homolog protein 1
MYH1	myosin. heavy chain 1. skeletal muscle. adult	*CDK4*	Cyclin-dependent kinase 4
MYH2	myosin. heavy chain 2. skeletal muscle. adult	*NOTCH2*	Neurogenic locus notch homolog protein 2
MYH4	myosin. heavy chain 4. skeletal muscle	*PDGFRA*	platelet-derived growth factor receptor. alpha polypeptide
FOXO1	Forkhead box O1	*MAP3K9*	mitogen-activated protein kinase kinase kinase 9
PHB	prohibitin	*IGF1R*	insulin-like growth factor 1 receptor
SPRY2	sprouty homolog 2	*ACVR1C*	activin A receptor. type IC
IGF1	insulin-like growth factor 1 (somatomedin C)	*GRB2*	growth factor receptor-bound protein 2
ST14	suppression of tumorigenicity 14 (colon carcinoma)	*CDC42*	cell division cycle 42 (GTP binding protein. 25 kDa)
CCNT1	cyclin T1	*OSCP1*	organic solute carrier partner 1
MMP13	matrix metallopeptidase 13 (collagenase 3)	*SOX2*	SRY (sex determining region Y)-box 2
CYP1B1	Cytochrome P450 1B1	*SP2*	Sp2 transcription factor
BCL6	B-cell CLL/lymphoma 6	*JUNB*	jun B proto-oncogene
SOCS1	suppressor of cytokine signaling 1	*JUND*	jun D proto-oncogene
SNAI1	snail homolog 1	*TGFB1*	transforming growth factor. beta 1
UBE2I	ubiquitin-conjugating enzyme E2I	*MECP2*	methyl CpG binding protein 2 (Rett syndrome)
KIT	V-Kit Hardy-Zuckerman 4 Feline Sarcoma Viral Oncogene Homolog	*TJP1*	tight junction protein 1
PDGFB	Platelet-derived growth factor beta polypeptide	*PTCH1*	patched 1
ATXN1	ataxin 1	*MEF2C*	myocyte enhancer factor 2C
BMPR2	bone morphogenetic protein receptor. type II (serine/threonine kinase)	*NFIA*	nuclear factor I/A
TLR2	toll-like receptor 2	*FOXO1*	forkhead box O1
CDKN1A	Cyclin-dependent kinase inhibitor 1A (p21. Cip1)	*PARP1*	poly (ADP-ribose) polymerase 1
RB1	Retinoblastoma 1	*RUNX1*	runt-related transcription factor 1
TCEAL1	transcription elongation factor A (SII)-like 1	*CALN1*	calneuron 1
BCL2L11	BCL2-like 11 (apoptosis facilitator)	*TGFBI*	transforming growth factor. beta-induced. 68 kDa

Table S4. Target Gene prediction of deregulated miRNAs.

miRNA	Target Gene
miR-937	CPOX. DCAF8. GLUL
miR-1303	SOAT. FHOD3. CDK6
miR-1908	COX2. COQ4. ATP6
miR-1915 *	PTCDI. WDR33
miR-762	AMD1
miR-379	IL11
miR-1286	U/I
miR-1275	U/I
miR-665	U/I
miR-602	U/I
miR-1248	U/I

* From miRTarBase prediction tool (less or no evidence).

Table S5. Summary of chromosomal loci of deregulated miRNAs in ES tissue.

Chromosomal Locus	miRNA	up/down
11q23.1	miR-34c-3p	down
11p15.5	miR-210	up
11q12.1	miR-130a	up
11p15.5	miR-675	down
11	miR-1908	down
22q13.31	LET-7B	up
22q13.1	miR-659	down
22	miR-1286	down
19p13.13	miR-27a	up
19p13.13	miR-23a	up
19p13.2	miR-199b-3p	up
19p13.2	miR-199a-5p	up
19p13.2	miR-638	down
19q13.32	miR-330-3p	down
19q13.41	LET-7E	up
17p13.1	miR-195	up
17p13.1	miR-497	up
17p13.3	miR-212	down
17q21.32	miR-196a	up
17q22	miR-301a	up
17q23.1	miR-21	up
9q22.32	LET-7F	up
9q22.32	miR-27b	up
9q22.32	LET-7A	up
9q34.3	miR-602	down
8q24.3	miR-937	down
8q24.22	miR-30b	up
8	miR-151-5p	up
1p36.22	miR-34a	up
1p34.2	miR-30e	up
1q32.1	miR-181a	up
1q32.1	miR-181b	up

Table S6. miRXplore™ Microarrays: 954 microRNAs probe-sequence.

Table S7. TaqMan® MicroRNA Human Assays form Applied biosystem used in this study.

miRNA	AB Assay ID	Target Sequence (5′–3′)
hsa-miR-181b	1098	AACAUUCAUUGCUGUCGGUGGG
hsa-miR-1915	121111_mat	CCCCAGGGCGACGCGGCGGG
hsa-miR-1275	2840	GUGGGGGAGAGGCUGUC
RNU6B	1093	CGCAAGGAUGACACGCAAAUUCGUGAAGCGUUCCAUAUUUUU
