# Supplementary material for: Ewing’s Sarcoma: An Analysis of miRNA Expression Profiles and Target Genes in Paraffin-Embedded Primary Tumor Tissue
Source: Int J Mol Sci. 2016 Apr 30;17(5):656. doi: 10.3390/ijms17050656 (PMC4881482; doi:10.3390/ijms17050656)
Supplement: Supplementary file 1 [file ijms-17-00656-s001.zip › ijms-126634-Supplementary Materials/Table S2 2016 Berardi.pdf]

id	GeneID	miRNA name	Exact Mann-Whitney U test		FC	>1.2	<-0.5
			uncorrected p-value	corrected p-value			
m_1	35816	LET-7B: HSA-LET-7B; MMU-LET-7B; RNO-LET-7B	0,0453604	0,26352255	1,700977572	1	0
m_2	35817	LET-7C: HSA-LET-7C; MMU-LET-7C; RNO-LET-7C	0,2100508	0,37319709	1,254791889	1	0
m_3	35830	MIR-125B: HSA-MIR-125B; MMU-MIR-125B-5P; RNO-MIR-125B-5P	0,134952	0,33830435	1,387844006	1	0
m_4	35837	MIR-130A: HSA-MIR-130A; MMU-MIR-130A; RNO-MIR-130A	0,0227743	0,20936437	1,802940185	1	0
m_5	35843	MIR-136: HSA-MIR-136; RNO-MIR-136; MMU-MIR-136	0,7368718	0,74501407	-0,639622245	0	1
m_6	35855	MIR-145: HSA-MIR-145; MMU-MIR-145; RNO-MIR-145	0,6273292	0,6413477	-1,105659124	0	1
m_7	35868	MIR-17: HSA-MIR-17; MMU-MIR-17; RNO-MIR-17 (RNO-MIR-17-5P)	0,1825711	0,35355029	1,214175661	1	0
m_8	35869	MIR-181A: HSA-MIR-181A; MMU-MIR-181A; RNO-MIR-181A	0,0227743	0,20936437	3,483396018	1	0
m_9	35879	MIR-195: HSA-MIR-195; MMU-MIR-195; RNO-MIR-195	0,0453604	0,26352255	2,364840938	1	0
m_10	35883	MIR-21: HSA-MIR-21; MMU-MIR-21; RNO-MIR-21	0,0133634	0,20936437	2,162445617	1	0
m_11	35884	MIR-210: HSA-MIR-210; MMU-MIR-210; RNO-MIR-210	0,0071523	0,20936437	2,037000568	1	0
m_12	35885	MIR-181A*: HSA-MIR-181A*; MMU-MIR-181A-1*; RNO-MIR-181A-1*	0,0811218	0,31253233	1,394557287	1	0
m_13	35887	MIR-23A: HSA-MIR-23A; MMU-MIR-23A; RNO-MIR-23A	0,0291737	0,20936437	1,557042028	1	0
m_14	35888	MIR-23B: HSA-MIR-23B; MMU-MIR-23B; RNO-MIR-23B	0,0969321	0,3167601	1,316642538	1	0
m_15	35893	MIR-27A: HSA-MIR-27A; MMU-MIR-27A; RNO-MIR-27A	0,0227743	0,20936437	1,646126413	1	0
m_16	35894	MIR-27B: HSA-MIR-27B; MMU-MIR-27B; RNO-MIR-27B	0,0291737	0,20936437	1,701162712	1	0
m_17	35899	MIR-30B: HSA-MIR-30B; MMU-MIR-30B; RNO-MIR-30B	0,0291737	0,20936437	2,119594446	1	0
m_18	35900	MIR-30C: HSA-MIR-30C; MMU-MIR-30C; RNO-MIR-30C	0,0453604	0,26352255	1,546223564	1	0
m_19	35910	MIR-361-5P: HSA-MIR-361-5P; MMU-MIR-361; RNO-MIR-361	0,0227743	0,20936437	1,221056398	1	0
m_20	35914	MIR-377: HSA-MIR-377; MMU-MIR-377; RNO-MIR-377	0,0811218	0,31253233	1,216463919	1	0
m_21	35922	MIR-425*: MMU-MIR-425*; HSA-MIR-425*	0,2405421	0,38783435	-0,556040759	0	1
m_22	35925	MIR-9*: MMU-MIR-9*; HSA-MIR-9*; RNO-MIR-9*	0,3470732	0,44107223	1,703389349	1	0
m_23	38314	LET-7A: HSA-LET-7A; MMU-LET-7A; RNO-LET-7A	0,0099755	0,20936437	2,259943742	1	0
m_24	38315	LET-7F: HSA-LET-7F; MMU-LET-7F; RNO-LET-7F	0,0133634	0,20936437	1,578072257	1	0
m_25	38316	MIR-100: HSA-MIR-100; MMU-MIR-100; RNO-MIR-100	0,0555242	0,27838153	1,245544862	1	0
m_26	38321	MIR-142-3P: HSA-MIR-142-3P; MMU-MIR-142-3P; RNO-MIR-142-3P	0,2732919	0,40660505	-0,770073055	0	1
m_27	38322	MIR-146B-5P: HSA-MIR-146B-5P; MMU-MIR-146B; RNO-MIR-146B	0,0133634	0,20936437	1,619529112	1	0
m_28	38325	MIR-199B-5P: HSA-MIR-199B-5P	0,0675701	0,29795977	1,751268151	1	0
m_29	38327	MIR-19B: HSA-MIR-19B; MMU-MIR-19B; RNO-MIR-19B	0,0365142	0,24298548	1,506461715	1	0
m_30	38335	MIR-219-5P: HSA-MIR-219-5P; MMU-MIR-219-5P; RNO-MIR-219-5P	0,3881046	0,45674052	-0,634035187	0	1
m_31	38337	MIR-221: HSA-MIR-221; MMU-MIR-221; RNO-MIR-221	0,0675701	0,29795977	3,147217506	1	0
m_32	38343	MIR-325-5P: HSA-MIR-325	0,1825711	0,35355029	-0,546802437	0	1
m_33	38345	MIR-330-3P: HSA-MIR-330-3P	0,0291737	0,20936437	-0,676200792	0	1
m_34	38349	MIR-365: HSA-MIR-365; MMU-MIR-365; RNO-MIR-365	0,0555242	0,27838153	-1,345631158	0	1
m_35	38351	MIR-369-3P: HSA-MIR-369-3P; MMU-MIR-369-3P; RNO-MIR-369-3P	0,0969321	0,3167601	-0,796789519	0	1
m_36	38365	MIR-429: HSA-MIR-429	0,3470732	0,44107223	1,20016811	1	0
m_37	38373	MIR-485-3P: HSA-MIR-485-3P; MMU-MIR-485*; RNO-MIR-485*	0,1146245	0,32521372	-0,831594715	0	1
m_38	38390	MIR-517C: HSA-MIR-517C	0,2100508	0,37319709	1,216104831	1	0
m_39	38396	MIR-524-5P: HSA-MIR-524-5P	0,3090533	0,42049627	-0,575801664	0	1
m_40	38412	MIR-562: HSA-MIR-562	0,134952	0,33830435	1,343073586	1	0
m_41	38413	MIR-563: HSA-MIR-563	0,134952	0,33830435	1,56818459	1	0
m_42	38420	MIR-572: HSA-MIR-572	0,0227743	0,20936437	-0,888176812	0	1
m_43	38427	MIR-583: HSA-MIR-583	0,134952	0,33830435	-1,076893445	0	1
m_44	38443	MIR-602: HSA-MIR-602	0,0291737	0,20936437	-0,836221025	0	1
m_45	38446	MIR-605: HSA-MIR-605	0,1146245	0,32521372	-0,598531834	0	1
m_46	38468	MIR-630: HSA-MIR-630	0,2405421	0,38783435	-0,721426487	0	1
m_47	38475	MIR-638: HSA-MIR-638	0,0099755	0,20936437	-1,077583316	0	1
m_48	38479	MIR-642A: HSA-MIR-642A	0,1146245	0,32521372	-1,121608947	0	1
m_49	38489	MIR-656: HSA-MIR-656	0,3881046	0,45674052	-0,733259004	0	1
m_50	38491	MIR-658: HSA-MIR-658	0,1573499	0,342798	-0,94260827	0	1
m_51	38492	MIR-659: HSA-MIR-659	0,0365142	0,24298548	-1,596659699	0	1
m_52	38493	MIR-661: HSA-MIR-661	0,3090533	0,42049627	-0,672613499	0	1
m_53	38499	MIR-106B: HSA-MIR-106B; MMU-MIR-106B; RNO-MIR-106B	0,0227743	0,20936437	1,374387113	1	0
m_54	38501	MIR-135A: HSA-MIR-135A; MMU-MIR-135A; RNO-MIR-135A	0,9109731	0,91097307	-0,63443027	0	1
m_55	38503	MIR-199A-5P: HSA-MIR-199A-5P; MMU-MIR-199A-5P; RNO-MIR-199A-5P	0,0291737	0,20936437	2,361398646	1	0
m_56	38517	MIR-296-5P: HSA-MIR-296-5P; MMU-MIR-296-5P; RNO-MIR-296*	0,5249388	0,5601388	-1,410379138	0	1
m_57	38528	MIR-379: HSA-MIR-379; MMU-MIR-379; RNO-MIR-379	0,0227743	0,20936437	1,446876012	1	0
m_58	38543	MIR-148B: HSA-MIR-148B; MMU-MIR-148B; RNO-MIR-148B-3P	0,1146245	0,32521372	1,350004104	1	0
m_59	38555	MIR-338-3P: RNO-MIR-338; MMU-MIR-338-3P; HSA-MIR-338-3P	0,0555242	0,27838153	1,221504962	1	0
m_60	38566	MIR-497: HSA-MIR-497; MMU-MIR-497; RNO-MIR-497	0,0227743	0,20936437	2,468076256	1	0
m_61	38569	MIR-29B: HSA-MIR-29B; MMU-MIR-29B; RNO-MIR-29B	0,0071523	0,20936437	2,937173211	1	0
m_62	38571	MIR-328-3P: HSA-MIR-328; MMU-MIR-328; RNO-MIR-328A	0,1573499	0,342798	-0,798686489	0	1
m_63	38613	MIR-550A: HSA-MIR-550A	0,0969321	0,3167601	-0,950888339	0	1
m_64	38616	MIR-556-5P: HSA-MIR-556-5P	0,6273292	0,6413477	-0,536668791	0	1
m_65	38630	MIR-624: HSA-MIR-624	0,0969321	0,3167601	-1,206551178	0	1
m_66	38631	MIR-625: HSA-MIR-625	0,6273292	0,6413477	-0,595109453	0	1
m_67	38635	MIR-636: HSA-MIR-636	0,0969321	0,3167601	-0,51221104	0	1
m_68	38641	MIR-655: HSA-MIR-655	0,3470732	0,44107223	-0,611472068	0	1
m_69	38643	MIR-663: HSA-MIR-663	0,0050819	0,20936437	-1,339518478	0	1
m_70	38654	KSHV-MIR-K12-10A: KSHV-MIR-K12-10A	0,1573499	0,342798	-0,810043295	0	1
m_71	38655	KSHV-MIR-K12-10B: KSHV-MIR-K12-10B	0,3090533	0,42049627	-0,690195236	0	1
m_72	38717	MIR-183: HSA-MIR-183; MMU-MIR-183; RNO-MIR-183	0,0453604	0,26352255	-1,263442073	0	1
m_73	38966	MIR-151-5P-151B: HSA-MIR-151-5P; MMU-MIR-151-5P; RNO-MIR-151;HSA-MIR-151B	0,0099755	0,20936437	1,690015675	1	0
m_74	38973	MIR-22*: RNO-MIR-22*; HSA-MIR-22*; MMU-MIR-22*	0,1146245	0,32521372	1,864098374	1	0
m_75	38983	MIR-301A: HSA-MIR-301A; MMU-MIR-301A; RNO-MIR-301A	0,0291737	0,20936437	1,260984605	1	0
m_76	39096	EBV-MIR-BART14-5P: EBV-MIR-BART14-5P	0,0365142	0,24298548	-0,62801174	0	1
m_77	39104	EBV-MIR-BART17-3P: EBV-MIR-BART17-3P	0,0811218	0,31253233	-1,002243603	0	1
m_78	39111	EBV-MIR-BART19-3P: EBV-MIR-BART19-3P	0,9109731	0,91097307	-0,936249145	0	1
m_79	39123	EBV-MIR-BART6-3P: EBV-MIR-BART6-3P	0,0227743	0,20936437	-0,843334524	0	1
m_80	39128	EBV-MIR-BART8-3P: EBV-MIR-BART8-3P	0,134952	0,33830435	-0,699428731	0	1
m_81	39132	EBV-MIR-BHRF1-1: EBV-MIR-BHRF1-1	0,0227743	0,20936437	-0,836378198	0	1
m_82	39138	HCMV-MIR-UL112: HCMV-MIR-UL112	0,3090533	0,42049627	-0,549304075	0	1
m_83	39139	HCMV-MIR-UL148D: HCMV-MIR-UL148D	0,0811218	0,31253233	-0,680659174	0	1
m_84	39149	HCMV-MIR-US33-5P: HCMV-MIR-US33-5P	0,1825711	0,35355029	-1,036001523	0	1
m_85	39153	LET-7D: HSA-LET-7D; MMU-LET-7D; RNO-LET-7D	0,0969321	0,3167601	1,226674804	1	0
m_86	39155	LET-7E: HSA-LET-7E; MMU-LET-7E; RNO-LET-7E	0,0291737	0,20936437	1,281646088	1	0
m_87	39156	LET-7G: HSA-LET-7G; MMU-LET-7G	0,0176925	0,20936437	1,627573169	1	0
m_88	39158	MIR-1A: HSA-MIR-1A; MMU-MIR-1A	0,8518727	0,85655336	1,94414027	1	0
m_89	39163	MIR-744: HSA-MIR-744; MMU-MIR-744; RNO-MIR-744	0,0969321	0,3167601	-0,85985332	0	1
m_90	39165	MIR-615-3P: RNO-MIR-615-3P; HSA-MIR-615-3P; MMU-MIR-615-3P	0,1573499	0,342798	-1,677181924	0	1
m_91	39166	MIR-615-5P: HSA-MIR-615-5P; MMU-MIR-615-5P; RNO-MIR-615	0,1573499	0,342798	-0,897136223	0	1
m_92	39175	MIR-885-3P: HSA-MIR-885-3P	0,0555242	0,27838153	-0,793522451	0	1
m_93	39185	MIR-874: HSA-MIR-874; MMU-MIR-874; RNO-MIR-874	0,3881046	0,45674052	-0,844128479	0	1
m_94	39196	MIR-665: HSA-MIR-665	0,0133634	0,20936437	-1,077518251	0	1
m_95	39199	MIR-106A: HSA-MIR-106A	0,0811218	0,31253233	1,402789294	1	0
m_96	39210	MIR-128: HSA-MIR-128; MMU-MIR-128; RNO-MIR-128	0,0365142	0,24298548	1,904723965	1	0
m_97	39211	MIR-133A: HSA-MIR-133A; MMU-MIR-133A; RNO-MIR-133A	0,3090533	0,42049627	4,802308539	1	0
m_98	39212	MIR-133B: HSA-MIR-133B; MMU-MIR-133B; RNO-MIR-133B	0,1825711	0,35355029	4,562390311	1	0
m_99	39216	MIR-138: HSA-MIR-138; MMU-MIR-138; RNO-MIR-138	0,4771316	0,52599445	1,732681807	1	0
m_100	39237	MIR-181B: HSA-MIR-181B; MMU-MIR-181B; RNO-MIR-181B	0,0071523	0,20936437	2,87116484	1	0
m_101	39243	MIR-185: HSA-MIR-185; MMU-MIR-185; RNO-MIR-185	0,0675701	0,29795977	-0,549278848	0	1
m_102	39249	MIR-18A*: HSA-MIR-18A*; MMU-MIR-18A*; RNO-MIR-18A*	0,3881046	0,45674052	-0,694048555	0	1
m_103	39254	MIR-191*: HSA-MIR-191*	0,0675701	0,29795977	-0,537511958	0	1
m_104	39256	MIR-193A-3P: HSA-MIR-193A-3P; MMU-MIR-193; RNO-MIR-193	0,7368718	0,74501407	-0,555269157	0	1
m_105	39260	MIR-196A: HSA-MIR-196A; MMU-MIR-196A; RNO-MIR-196A	0,0050819	0,20936437	1,723288209	1	0
m_106	39264	MIR-198: HSA-MIR-198	0,1573499	0,342798	-0,61885019	0	1
m_107	39265	MIR-199A-3P-199B-3P: HSA-MIR-199A-3P; HSA-MIR-199B-3P; MMU-MIR-199A-3P; MMU-MIR-199B; RNO-MIR-199A-3P	0,0176925	0,20936437	2,607320218	1	0
m_108	39266	MIR-891A: HSA-MIR-891A	0,2405421	0,38783435	-0,655585085	0	1
m_109	39270	MIR-892B: HSA-MIR-892B	0,1825711	0,35355029	-1,032215193	0	1
m_110	39278	MIR-212: HSA-MIR-212; MMU-MIR-212-3P; RNO-MIR-212	0,0071523	0,20936437	-1,386056739	0	1
m_111	39280	MIR-214: HSA-MIR-214; MMU-MIR-214; RNO-MIR-214	0,0555242	0,27838153	1,799970622	1	0
m_112	39288	MIR-222: HSA-MIR-222; MMU-MIR-222; RNO-MIR-222	0,0453604	0,26352255	2,160497442	1	0
m_113	39291	MIR-223: HSA-MIR-223; MMU-MIR-223; RNO-MIR-223	0,0133634	0,20936437	-1,610242879	0	1
m_114	39294	MIR-26A: HSA-MIR-26A; MMU-MIR-26A; RNO-MIR-26A	0,1573499	0,342798	1,283564621	1	0
m_115	39297	MIR-296-3P: HSA-MIR-296-3P; MMU-MIR-296-3P; RNO-MIR-296	0,2405421	0,38783435	-0,591377027	0	1
m_116	39298	MIR-298: HSA-MIR-298	0,1573499	0,342798	-0,701295113	0	1
m_117	39299	MIR-29A: HSA-MIR-29A; MMU-MIR-29A; RNO-MIR-29A	0,0071523	0,20936437	3,211108838	1	0
m_118	39305	MIR-302C*: HSA-MIR-302C*	0,5249388	0,5601388	-0,50659058	0	1
m_119	39307	MIR-30E: MMU-MIR-30E; RNO-MIR-30E; HSA-MIR-30E	0,0176925	0,20936437	1,284191047	1	0
m_120	39311	MIR-32: HSA-MIR-32; MMU-MIR-32; RNO-MIR-32	0,5753812	0,60340832	-0,558283107	0	1
m_121	39335	MIR-34A: HSA-MIR-34A; MMU-MIR-34A; RNO-MIR-34A	0,0291737	0,20936437	2,602895388	1	0
m_122	39343	MIR-370: HSA-MIR-370; MMU-MIR-370; RNO-MIR-370	0,134952	0,33830435	-1,372667063	0	1
m_123	39346	MIR-371-5P: HSA-MIR-371-5P	0,2405421	0,38783435	-1,220825058	0	1
m_124	39347	MIR-373*: HSA-MIR-373*	0,3470732	0,44107223	-0,680808188	0	1
m_125	39348	MIR-376C: HSA-MIR-376C (HSA-MIR-368)	0,0099755	0,20936437	1,391755139	1	0
m_126	39353	MIR-384-3P: HSA-MIR-384 (HSA-MIR-384-3P)	0,6273292	0,6413477	-0,584294998	0	1
m_127	39354	MIR-384-5P: HSA-MIR-384-5P	0,2732919	0,40660505	-0,668053639	0	1
m_128	39356	MIR-409-3P: HSA-MIR-409-3P; RNO-MIR-409-3P; MMU-MIR-409-3P	0,2100508	0,37319709	-0,59034474	0	1
m_129	39369	MIR-450B-5P: HSA-MIR-450B-5P	0,7368718	0,74501407	-0,528895826	0	1
m_130	39377	MIR-455-3P: HSA-MIR-455-3P	0,0555242	0,27838153	1,367296362	1	0
m_131	39380	MIR-483-3P: HSA-MIR-483-3P	0,4771316	0,52599445	-1,015314097	0	1
m_132	39382	MIR-483-5P: HSA-MIR-483-5P	0,0811218	0,31253233	-0,918835879	0	1
m_133	39389	MIR-491-5P: HSA-MIR-491-5P; MMU-MIR-491	0,4312065	0,48560482	-1,520532442	0	1
m_134	39393	MIR-493*: HSA-MIR-493*; MMU-MIR-493*	0,1146245	0,32521372	1,488125561	1	0
m_135	39407	MIR-508-5P: HSA-MIR-508-5P	0,3881046	0,45674052	-0,946576648	0	1
m_136	39435	MIR-518C*: HSA-MIR-518C*	0,2405421	0,38783435	-0,71278064	0	1
m_137	39445	MIR-518F: HSA-MIR-518F	0,3881046	0,45674052	-0,574214016	0	1
m_138	39454	MIR-519E*: HSA-MIR-519E*	0,4771316	0,52599445	-0,758811238	0	1
m_139	39459	MIR-520D-5P: HSA-MIR-520D-5P	0,2100508	0,37319709	-1,151134948	0	1
m_140	39476	MIR-526B: HSA-MIR-526B	0,3881046	0,45674052	-0,923623818	0	1
m_141	39480	MIR-518A-5P-527_2: HSA-MIR-518A-5P; HSA-MIR-527	0,0969321	0,3167601	-1,071195247	0	1
m_142	39892	MIR-769-3P: HSA-MIR-769-3P	0,134952	0,33830435	-0,807305293	0	1
m_143	39894	MIR-765: HSA-MIR-765	0,0555242	0,27838153	-0,807067852	0	1
m_144	39901	MIR-143: HSA-MIR-143; MMU-MIR-143; RNO-MIR-143	0,5753812	0,60340832	-0,577815599	0	1
m_145	39903	MIR-675: HSA-MIR-675	0,0176925	0,20936437	-1,219277168	0	1
m_146	39904	SV40-MIR-S1-5P: SV40-MIR-S1-5P	0,0071523	0,20936437	-1,648753975	0	1
m_147	39914	MIR-193A-5P: HSA-MIR-193A-5P	0,1573499	0,342798	-0,957385474	0	1
m_148	39933	MIR-548A-5P: HSA-MIR-548A-5P	0,3090533	0,42049627	-0,64762886	0	1
m_149	39934	MIR-548B-5P: HSA-MIR-548B-5P	0,4771316	0,52599445	-0,521094231	0	1
m_150	39939	MIR-338-5P: HSA-MIR-338-5P; MMU-MIR-338-5P; RNO-MIR-338*	0,5753812	0,60340832	-0,519109635	0	1
m_151	39943	EBV-MIR-BART13*: EBV-MIR-BART13*	0,3090533	0,42049627	-0,982518546	0	1
m_152	39944	MIR-122*: HSA-MIR-122*; MMU-MIR-122*	0,5249388	0,5601388	-0,708950158	0	1
m_153	39946	MIR-940: HSA-MIR-940	0,1146245	0,32521372	-1,04096132	0	1
m_154	39947	MIR-148B*: HSA-MIR-148B*; MMU-MIR-148B*	0,4312065	0,48560482	-0,576618298	0	1
m_155	39951	MIR-34C-3P: HSA-MIR-34C-3P	0,0071523	0,20936437	-1,780710627	0	1
m_156	39957	MIR-125A-3P: HSA-MIR-125A-3P; MMU-MIR-125A-3P; RNO-MIR-125A-3P	0,1825711	0,35355029	-1,758027896	0	1
m_157	39958	MIR-936: HSA-MIR-936	0,1573499	0,342798	-1,897980164	0	1
m_158	39960	KSHV-MIR-K12-9*: KSHV-MIR-K12-9*	0,134952	0,33830435	-0,747772434	0	1
m_159	39965	MIR-125B-1*: HSA-MIR-125B-1*; MMU-MIR-125B-1-3P; RNO-MIR-125B-3P	0,3881046	0,45674052	-1,285662928	0	1
m_160	39981	MIR-219-2-3P: HSA-MIR-219-2-3P; RNO-MIR-219-2-3P	0,3090533	0,42049627	-0,709197016	0	1
m_161	39982	MIR-27B*: HSA-MIR-27B*; MMU-MIR-27B*; RNO-MIR-27B*	0,4312065	0,48560482	-0,578161916	0	1
m_162	39985	MIR-219-1-3P: HSA-MIR-219-1-3P	0,2405421	0,38783435	-0,800695795	0	1
m_163	39988	MIR-25*: HSA-MIR-25*; MMU-MIR-25*	0,5753812	0,60340832	-0,984972239	0	1
m_164	39989	MIR-92B*: HSA-MIR-92B*; RNO-MIR-92B*	0,1825711	0,35355029	-1,081236788	0	1
m_165	39990	MIR-149*: HSA-MIR-149*	0,0969321	0,3167601	-1,03774565	0	1
m_166	39992	MIR-185*: HSA-MIR-185*; MMU-MIR-185*	0,3881046	0,45674052	-0,896289741	0	1
m_167	39994	MIR-342-5P: MMU-MIR-342-5P; RNO-MIR-342-5P; HSA-MIR-342-5P	0,3090533	0,42049627	-1,805185971	0	1
m_168	40006	MIR-937: HSA-MIR-937	0,0453604	0,26352255	-2,295916752	0	1
m_169	40007	MIR-628-5P: HSA-MIR-628-5P	0,4312065	0,48560482	-0,539201663	0	1
m_170	40008	MIR-218-1*: HSA-MIR-218-1*	0,4771316	0,52599445	-0,57936	0	1
m_171	40012	MIR-322*-MIR-424*: HSA-MIR-424* (HSA-MIR-322*)	0,5249388	0,5601388	-0,520334499	0	1
m_172	40018	MIR-99A*: HSA-MIR-99A*	0,2100508	0,37319709	-0,949522328	0	1
m_173	40022	MIR-34B-3P: HSA-MIR-34B; MMU-MIR-34B-3P	0,4312065	0,48560482	-0,617093334	0	1
m_174	40032	MIR-33B*: HSA-MIR-33B*	0,2405421	0,38783435	-0,694308556	0	1
m_175	40039	MIR-218-2*: HSA-MIR-218-2*; MMU-MIR-218-2*; RNO-MIR-218A-2*	0,3090533	0,42049627	-0,694214275	0	1
m_176	40040	MIR-16-2*: HSA-MIR-16-2*	0,1825711	0,35355029	-0,819239811	0	1
m_177	40041	MIR-195*: HSA-MIR-195*; RNO-MIR-195*	0,5249388	0,5601388	-0,671589363	0	1
m_178	40044	MIR-194*: HSA-MIR-194*; RNO-MIR-194*	0,2732919	0,40660505	-1,604405732	0	1
m_179	40045	MIR-935: HSA-MIR-935; RNO-MIR-935	0,5249388	0,5601388	-0,604751961	0	1
m_180	40051	MIR-26A-2*: HSA-MIR-26A-2*	0,3090533	0,42049627	-0,75639491	0	1
m_181	40052	MIR-26A-1*: HSA-MIR-26A-1*; RNO-MIR-26A*	0,4312065	0,48560482	-0,574436674	0	1
m_182	40053	MIR-146A*: HSA-MIR-146A*	0,6273292	0,6413477	-0,537625912	0	1
m_183	40057	MIR-15B*: HSA-MIR-15B*; MMU-MIR-15B*	0,5249388	0,5601388	-0,681255119	0	1
m_184	40064	MIR-193B*: HSA-MIR-193B*	0,4771316	0,52599445	-0,575692755	0	1
m_185	40067	MIR-124*: HSA-MIR-124*; MMU-MIR-124*; RNO-MIR-124*	0,2732919	0,40660505	-0,879373445	0	1
m_186	40068	MIR-921: HSA-MIR-921	0,5249388	0,5601388	-0,798324364	0	1
m_187	40069	LET-7B*: HSA-LET-7B*; MMU-LET-7B*; RNO-LET-7B*	0,3881046	0,45674052	-0,61065939	0	1
m_188	40073	LET-7E*: HSA-LET-7E*; RNO-LET-7E*	0,4312065	0,48560482	-0,508164071	0	1
m_189	40079	MIR-127*: HSA-MIR-127-5P; MMU-MIR-127*; RNO-MIR-127*	0,6273292	0,6413477	-1,098888943	0	1
m_190	40084	MIR-30C-2*: HSA-MIR-30C-2*; MMU-MIR-30C-2*; RNO-MIR-30C-2*	0,2100508	0,37319709	-1,055329461	0	1
m_191	40085	MIR-30C-1*: HSA-MIR-30C-1*; MMU-MIR-30C-1*; RNO-MIR-30C-1*	0,1825711	0,35355029	-1,161140005	0	1
m_192	40086	MIR-30B*: HSA-MIR-30B*	0,134952	0,33830435	-0,820597697	0	1
m_193	40088	MIR-150*: HSA-MIR-150*; RNO-MIR-150*	0,3090533	0,42049627	-0,660919629	0	1
m_194	40095	MIR-374A*: HSA-MIR-374A*	0,5249388	0,5601388	-0,501718009	0	1
m_195	40097	MIR-551B*: HSA-MIR-551B*	0,1573499	0,342798	-1,096175611	0	1
m_196	40106	MIR-138-1*: HSA-MIR-138-1*	0,3470732	0,44107223	-0,902869386	0	1
m_197	40113	MIR-139-3P: HSA-MIR-139-3P; MMU-MIR-139-3P; RNO-MIR-139-3P	0,3090533	0,42049627	-1,11132731	0	1
m_198	40114	MIR-144*: HSA-MIR-144*; MMU-MIR-144*; RNO-MIR-144*	0,6273292	0,6413477	-0,56336308	0	1
m_199	40117	MIR-187*: HSA-MIR-187*; MMU-MIR-187*; RNO-MIR-187*	0,2405421	0,38783435	-0,630644617	0	1
m_200	40118	MIR-505*: HSA-MIR-505*; MMU-MIR-505-5P; RNO-MIR-505*	0,6273292	0,6413477	-1,289515735	0	1
m_201	40120	MIR-23A*: HSA-MIR-23A*; RNO-MIR-23A*	0,3090533	0,42049627	-0,936791108	0	1
m_202	40121	MIR-92A-2*: HSA-MIR-92A-2*	0,2732919	0,40660505	-0,919409491	0	1
m_203	40122	MIR-143*: HSA-MIR-143*; MMU-MIR-143*; RNO-MIR-143*	0,2100508	0,37319709	-1,086568949	0	1
m_204	40124	MIR-183*: HSA-MIR-183*; MMU-MIR-183*; RNO-MIR-183*	0,1573499	0,342798	-1,150215084	0	1
m_205	40127	EBV-MIR-BART9*: EBV-MIR-BART9*	0,1573499	0,342798	-0,971266564	0	1
m_206	40132	MIR-135A*: HSA-MIR-135A*; MMU-MIR-135A-1*	0,2405421	0,38783435	-1,174484405	0	1
m_207	40134	MIR-379*: HSA-MIR-379*; RNO-MIR-379*	0,3470732	0,44107223	-0,585839038	0	1
m_208	40135	MIR-125B-2*: HSA-MIR-125B-2*	0,4312065	0,48560482	-0,675100841	0	1
m_209	40136	MIR-545*: HSA-MIR-545*	0,1573499	0,342798	-0,864315149	0	1
m_210	40139	MIR-361-3P: HSA-MIR-361-3P; MMU-MIR-361*	0,1573499	0,342798	-0,848865903	0	1
m_211	40143	MIR-942: HSA-MIR-942	0,2405421	0,38783435	-0,918917705	0	1
m_212	40146	MIR-18B*: HSA-MIR-18B*; MMU-MIR-18B*	0,0453604	0,26352255	-1,110742814	0	1

m_213	40147	MIR-146B-3P: HSA-MIR-146B-3P	0,134952	0,33830435	-1,067240734	0	1
m_214	40148	MIR-938: HSA-MIR-938	0,0969321	0,3167601	-1,844452169	0	1
m_215	40157	MIR-939: HSA-MIR-939	0,1573499	0,342798	-0,709645997	0	1
m_216	40160	MIR-23B*: HSA-MIR-23B*; MMU-MIR-23B*; RNO-MIR-23B*	0,2732919	0,40660505	-0,738348225	0	1
m_217	40163	MIR-877*: MMU-MIR-877*; HSA-MIR-877*	0,0811218	0,31253233	-1,118528227	0	1
m_218	40164	MIR-934: HSA-MIR-934	0,3090533	0,42049627	-0,601753078	0	1
m_219	40165	MIR-593: HSA-MIR-593	0,2405421	0,38783435	-0,780644626	0	1
m_220	40166	MIR-933: HSA-MIR-933	0,1573499	0,342798	-1,135396139	0	1
m_221	40167	MIR-130A*: HSA-MIR-130A*; RNO-MIR-130A*	0,3470732	0,44107223	-0,792223193	0	1
m_222	40169	MIR-450B-3P: HSA-MIR-450B-3P	0,2732919	0,40660505	-0,67837119	0	1
m_223	40171	HCMV-MIR-UL36*: HCMV-MIR-UL36*	0,3881046	0,45674052	-0,749767056	0	1
m_224	40172	MIR-335*: HSA-MIR-335*; MMU-MIR-335-3P	0,1825711	0,35355029	-1,022487466	0	1
m_225	40194	MIR-486-3P: HSA-MIR-486-3P; MMU-MIR-3107*	0,1825711	0,35355029	-0,874269683	0	1
m_226	40195	MIR-625*: HSA-MIR-625*	0,0675701	0,29795977	-1,571166517	0	1
m_227	40200	MIR-328-5P: MMU-MIR-328*; HSA-MIR-328-5P; RNO-MIR-328A*	0,2405421	0,38783435	-0,685588859	0	1
m_228	40214	MIR-1224-3P: HSA-MIR-1224-3P	0,1825711	0,35355029	-0,870157204	0	1
m_229	40215	MIR-1224-5P: HSA-MIR-1224-5P	0,2732919	0,40660505	-0,729046626	0	1
m_230	40216	MIR-1225-3P: HSA-MIR-1225-3P	0,0675701	0,29795977	-1,070154931	0	1
m_231	40217	MIR-1225-5P: HSA-MIR-1225-5P	0,3090533	0,42049627	-1,234142885	0	1
m_232	40218	MIR-1226: HSA-MIR-1226	0,1825711	0,35355029	-0,809912142	0	1
m_233	40219	MIR-1226*: HSA-MIR-1226*	0,2732919	0,40660505	-0,528439957	0	1
m_234	40221	MIR-1228: HSA-MIR-1228	0,0811218	0,31253233	-0,86675396	0	1
m_235	40222	MIR-1228*: HSA-MIR-1228*	0,0227743	0,20936437	-1,25250658	0	1
m_236	40223	MIR-1229: HSA-MIR-1229	0,134952	0,33830435	-1,027328834	0	1
m_237	40225	MIR-1233: HSA-MIR-1233	0,134952	0,33830435	-0,756917594	0	1
m_238	40226	MIR-1234: HSA-MIR-1234	0,1146245	0,32521372	-0,65169462	0	1
m_239	40227	MIR-1236: HSA-MIR-1236	0,0675701	0,29795977	-0,975755663	0	1
m_240	40228	MIR-1237: HSA-MIR-1237	0,1146245	0,32521372	-1,1019558	0	1
m_241	40231	MIR-513C: HSA-MIR-513C	0,8518727	0,85655336	-0,750787371	0	1
m_242	40285	HIV1-MIR-TAR-3P: HIV1-MIR-TAR-3P	0,0811218	0,31253233	-2,284008748	0	1
m_243	40286	HIV1-MIR-TAR-5P: HIV1-MIR-TAR-5P	0,2100508	0,37319709	-0,799725	0	1
m_244	40289	MIR-1180: HSA-MIR-1180	0,2732919	0,40660505	-0,638587605	0	1
m_245	40290	MIR-1181: HSA-MIR-1181	0,134952	0,33830435	-0,876054174	0	1
m_246	40291	MIR-1182: HSA-MIR-1182	0,3470732	0,44107223	-0,849646133	0	1
m_247	40292	MIR-1183: HSA-MIR-1183	0,4312065	0,48560482	-0,835804648	0	1
m_248	40293	MIR-1184: HSA-MIR-1184	0,1825711	0,35355029	-0,890305696	0	1
m_249	40294	MIR-1185: HSA-MIR-1185	0,134952	0,33830435	-1,015419214	0	1
m_250	40305	MIR-1197: HSA-MIR-1197; MMU-MIR-1197	0,134952	0,33830435	-0,763695657	0	1
m_251	40308	MIR-1200: HSA-MIR-1200	0,1825711	0,35355029	-0,952500287	0	1
m_252	40310	MIR-1202: HSA-MIR-1202	0,0675701	0,29795977	-1,935084668	0	1
m_253	40311	MIR-1203: HSA-MIR-1203	0,3881046	0,45674052	-0,890992535	0	1
m_254	40312	MIR-1204: HSA-MIR-1204	0,1146245	0,32521372	-1,19944221	0	1
m_255	40313	MIR-1205: HSA-MIR-1205	0,2100508	0,37319709	-0,840352594	0	1
m_256	40314	MIR-1206: HSA-MIR-1206	0,2732919	0,40660505	-0,526492654	0	1
m_257	40315	MIR-1207-3P: HSA-MIR-1207-3P	0,3090533	0,42049627	-0,66827805	0	1
m_258	40316	MIR-1207-5P: HSA-MIR-1207-5P	0,0675701	0,29795977	-1,006156146	0	1
m_259	40317	MIR-1208: HSA-MIR-1208	0,2405421	0,38783435	-0,661825224	0	1
m_260	40318	MIR-1243: HSA-MIR-1243	0,3470732	0,44107223	-0,678155647	0	1
m_261	40320	MIR-1245: HSA-MIR-1245	0,2405421	0,38783435	-0,73680602	0	1
m_262	40322	MIR-1247: HSA-MIR-1247	0,1825711	0,35355029	-0,782645004	0	1
m_263	40323	MIR-1248: HSA-MIR-1248	0,0291737	0,20936437	2,026344903	1	0
m_264	40324	MIR-1249: HSA-MIR-1249	0,0555242	0,27838153	-1,22502147	0	1
m_265	40325	MIR-1250: HSA-MIR-1250	0,3470732	0,44107223	-0,671572391	0	1
m_266	40327	MIR-1252: HSA-MIR-1252	0,4771316	0,52599445	-0,531078645	0	1
m_267	40328	MIR-1253: HSA-MIR-1253	0,3470732	0,44107223	-1,260496087	0	1
m_268	40329	MIR-1254: HSA-MIR-1254	0,1825711	0,35355029	-0,942244827	0	1
m_269	40332	MIR-1256: HSA-MIR-1256	0,2732919	0,40660505	-0,939694043	0	1
m_270	40333	MIR-1257: HSA-MIR-1257	0,2732919	0,40660505	-0,707858026	0	1
m_271	40334	MIR-1258: HSA-MIR-1258	0,0969321	0,3167601	-1,162053675	0	1
m_272	40341	MIR-1265: HSA-MIR-1265	0,3470732	0,44107223	-0,54866861	0	1
m_273	40343	MIR-1267: HSA-MIR-1267	0,0675701	0,29795977	-1,241243213	0	1
m_274	40344	MIR-1268-1268B: HSA-MIR-1268; HSA-MIR-1268B	0,0811218	0,31253233	-0,97581086	0	1
m_275	40346	MIR-1270: HSA-MIR-1270	0,3090533	0,42049627	-0,640819412	0	1
m_276	40348	MIR-1272: HSA-MIR-1272	0,2405421	0,38783435	-0,538751543	0	1
m_277	40349	MIR-1273: HSA-MIR-1273	0,1825711	0,35355029	-0,804193867	0	1
m_278	40352	MIR-1275: HSA-MIR-1275	0,0291737	0,20936437	-1,668322453	0	1
m_279	40353	MIR-1276: HSA-MIR-1276	0,5249388	0,5601388	-0,532490422	0	1
m_280	40354	MIR-1277: HSA-MIR-1277	0,3090533	0,42049627	-0,715158934	0	1
m_281	40355	MIR-1278: HSA-MIR-1278	0,1146245	0,32521372	-0,75148565	0	1
m_282	40356	MIR-1279: HSA-MIR-1279	0,4312065	0,48560482	-0,6691414	0	1
m_283	40358	MIR-1281: HSA-MIR-1281	0,0555242	0,27838153	-1,094332671	0	1
m_284	40359	MIR-1282: HSA-MIR-1282	0,0969321	0,3167601	-0,855602402	0	1
m_285	40360	MIR-1283: HSA-MIR-1283	0,2405421	0,38783435	-0,596900989	0	1
m_286	40361	MIR-1284: HSA-MIR-1284	0,2100508	0,37319709	-1,242613373	0	1
m_287	40362	MIR-1285: HSA-MIR-1285	0,2100508	0,37319709	-0,626898125	0	1
m_288	40363	MIR-1286: HSA-MIR-1286	0,0291737	0,20936437	-1,495022244	0	1
m_289	40365	MIR-1288: HSA-MIR-1288	0,2100508	0,37319709	-0,719815813	0	1
m_290	40366	MIR-1289: HSA-MIR-1289	0,2405421	0,38783435	-0,574656546	0	1
m_291	40368	MIR-1291: HSA-MIR-1291	0,6273292	0,6413477	-0,60798053	0	1
m_292	40369	MIR-1292: HSA-MIR-1292	0,2100508	0,37319709	-0,682532082	0	1
m_293	40370	MIR-1293: HSA-MIR-1293	0,1573499	0,342798	-1,110484566	0	1
m_294	40371	MIR-1294: HSA-MIR-1294	0,2100508	0,37319709	-0,663874332	0	1
m_295	40372	MIR-1295: HSA-MIR-1295	0,4312065	0,48560482	-0,591303452	0	1
m_296	40373	MIR-1296: HSA-MIR-1296	0,0969321	0,3167601	-1,29527135	0	1
m_297	40376	MIR-1299: HSA-MIR-1299	0,1825711	0,35355029	-0,852126594	0	1
m_298	40379	MIR-1301: HSA-MIR-1301	0,3881046	0,45674052	-1,228081046	0	1
m_299	40381	MIR-1303: HSA-MIR-1303	0,0176925	0,20936437	-1,319485649	0	1
m_300	40382	MIR-1304: HSA-MIR-1304	0,1573499	0,342798	-0,739576579	0	1
m_301	40383	MIR-1305: HSA-MIR-1305	0,0969321	0,3167601	-1,131821081	0	1
m_302	40385	MIR-1307: HSA-MIR-1307	0,3881046	0,45674052	-0,711869306	0	1
m_303	40387	MIR-1321: HSA-MIR-1321	0,3881046	0,45674052	-0,605792634	0	1
m_304	40389	MIR-1323: HSA-MIR-1323	0,1573499	0,342798	-1,205033003	0	1
m_305	40390	MIR-1324: HSA-MIR-1324	0,3881046	0,45674052	-0,576769037	0	1
m_306	40391	MIR-1825: HSA-MIR-1825	0,0969321	0,3167601	-1,155483632	0	1
m_307	40393	MIR-1827: HSA-MIR-1827	0,3881046	0,45674052	-0,565669993	0	1
m_308	40395	MIR-302F: HSA-MIR-302F	0,3881046	0,45674052	-0,561444136	0	1
m_309	40398	MIR-320D: HSA-MIR-320D	0,0099755	0,20936437	1,298152279	1	0
m_310	40405	MIR-548F-MIR-548G: HSA-MIR-548F; HSA-MIR-548G	0,4312065	0,48560482	-0,666835523	0	1
m_311	40412	MIR-548N: HSA-MIR-548N	0,2405421	0,38783435	-0,601294457	0	1
m_312	40413	MIR-548O: HSA-MIR-548O	0,3090533	0,42049627	-0,565124978	0	1
m_313	40414	MIR-548P-548AM: HSA-MIR-548P; HSA-MIR-548AM	0,3470732	0,44107223	-0,639475662	0	1
m_314	40416	MIR-664-3P: HSA-MIR-664	0,2405421	0,38783435	-0,646083034	0	1
m_315	40427	MIR-103B: HSA-MIR-103B	0,3470732	0,44107223	-0,507613681	0	1
m_316	40429	MIR-1469: HSA-MIR-1469	0,1573499	0,342798	-0,848669786	0	1
m_317	40430	MIR-1470: HSA-MIR-1470	0,0811218	0,31253233	-0,927090389	0	1
m_318	40431	MIR-1471: HSA-MIR-1471	0,1146245	0,32521372	-2,385371601	0	1
m_319	40433	MIR-1538: HSA-MIR-1538	0,2732919	0,40660505	-0,677709287	0	1
m_320	40434	MIR-1539: HSA-MIR-1539	0,3470732	0,44107223	-0,701775593	0	1
m_321	40435	MIR-1908: HSA-MIR-1908	0,0133634	0,20936437	-1,712608661	0	1
m_322	40436	MIR-1909*: HSA-MIR-1909*	0,0969321	0,3167601	-0,667943091	0	1
m_323	40437	MIR-1909: HSA-MIR-1909	0,5753812	0,60340832	-0,940424887	0	1
m_324	40438	MIR-1910: HSA-MIR-1910	0,2732919	0,40660505	-0,754550044	0	1
m_325	40440	MIR-1911*: HSA-MIR-1911*	0,4312065	0,48560482	-0,544863646	0	1
m_326	40442	MIR-1913: HSA-MIR-1913	0,1146245	0,32521372	-1,240608251	0	1
m_327	40443	MIR-1914: HSA-MIR-1914	0,2100508	0,37319709	-0,837384089	0	1
m_328	40444	MIR-1914*: HSA-MIR-1914*	0,134952	0,33830435	-1,708591822	0	1
m_329	40445	MIR-1915*: HSA-MIR-1915*	0,0453604	0,26352255	-1,26347519	0	1
m_330	40446	MIR-1915: HSA-MIR-1915	0,0099755	0,20936437	-1,609043615	0	1
m_331	40447	MIR-675*: HSA-MIR-675*	0,2732919	0,40660505	-0,601867056	0	1
m_332	40466	HSV1-MIR-H2*: HSV1-MIR-H2*	0,3881046	0,45674052	-0,695118702	0	1
m_333	40467	HSV1-MIR-H2: HSV1-MIR-H2	0,3470732	0,44107223	-0,76288025	0	1
m_334	40472	HSV1-MIR-H6-3P: HSV1-MIR-H6-3P	0,3090533	0,42049627	-0,911146291	0	1
m_335	40473	JCV-MIR-J1-5P: JCV-MIR-J1-5P	0,5249388	0,5601388	-1,172414012	0	1
m_336	40475	BKV-MIR-B1-5P: BKV-MIR-B1-5P	0,3470732	0,44107223	-1,064258108	0	1
m_337	40476	LET-7A-2*: HSA-LET-7A-2*; MMU-LET-7A-2*	0,3881046	0,45674052	-0,587605048	0	1
m_338	40477	MIR-103A-2*: HSA-MIR-103A-2*; RNO-MIR-103-2*; MMU-MIR-103-2*	0,3470732	0,44107223	-0,579490868	0	1
m_339	40490	MIR-2054: HSA-MIR-2054	0,4312065	0,48560482	-0,532974574	0	1
m_340	40491	MIR-2110: HSA-MIR-2110	0,3090533	0,42049627	-1,402819831	0	1
m_341	40492	MIR-2113: HSA-MIR-2113	0,6815358	0,69482484	-1,029590532	0	1
m_342	40493	MIR-224*: HSA-MIR-224*; MMU-MIR-224*; RNO-MIR-224*	0,0969321	0,3167601	-1,604071474	0	1
m_343	40494	MIR-365-2*: HSA-MIR-365*; MMU-MIR-365-2*; RNO-MIR-365*	0,1573499	0,342798	-0,838988401	0	1
m_344	40555	EBV-MIR-BART21-3P: EBV-MIR-BART21-3P	0,3090533	0,42049627	-0,893133491	0	1
m_345	40556	EBV-MIR-BART21-5P: EBV-MIR-BART21-5P	0,2732919	0,40660505	-0,665269723	0	1
m_346	40557	EBV-MIR-BART22: EBV-MIR-BART22	0,3470732	0,44107223	-0,539289302	0	1
m_347	40559	EBV-MIR-BART5*: EBV-MIR-BART5*	0,2732919	0,40660505	-0,842738626	0	1
m_348	40560	HSV2-MIR-H2: HSV2-MIR-H2	0,2732919	0,40660505	-1,90890616	0	1
m_349	40561	HSV2-MIR-H3: HSV2-MIR-H3	0,1825711	0,35355029	-1,567671265	0	1
m_350	40564	MCV-MIR-M1-3P: MCV-MIR-M1-3P	0,1825711	0,35355029	-0,98566544	0	1
m_351	40565	MCV-MIR-M1-5P: MCV-MIR-M1-5P	0,3881046	0,45674052	-0,654695755	0	1
m_352	41412	HBV-MIR-B20: HBV-MIR-B20	0,1146245	0,32521372	-0,661282315	0	1
m_353	41413	HBV-MIR-B2RC: HBV-MIR-B2RC	0,2405421	0,38783435	-0,691317039	0	1
m_354	41414	HBV-MIR-B4: HBV-MIR-B4	0,3881046	0,45674052	-0,674515843	0	1
m_355	41416	MIR-2114*: HSA-MIR-2114*	0,5753812	0,60340832	-0,647927141	0	1
m_356	41417	MIR-2115: HSA-MIR-2115	0,3881046	0,45674052	-0,797501136	0	1
m_357	41418	MIR-2115*: HSA-MIR-2115*	0,2100508	0,37319709	-0,594206116	0	1
m_358	41419	MIR-2116: HSA-MIR-2116	0,0555242	0,27838153	-0,93745772	0	1
m_359	41424	MIR-2278: HSA-MIR-2278	0,2100508	0,37319709	-0,689465128	0	1
m_360	41429	MIR-711: HSA-MIR-711	0,1146245	0,32521372	-0,817655005	0	1
m_361	41430	MIR-718: HSA-MIR-718	0,134952	0,33830435	-0,584197887	0	1
m_362	41431	MIR-762: HSA-MIR-762	0,0133634	0,20936437	-0,965705224	0	1
m_363	41433	HSV1-MIR-H7: HSV1-MIR-H7	0,1146245	0,32521372	-0,839125849	0	1
m_364	41434	HSV1-MIR-H7*: HSV1-MIR-H7*	0,0227743	0,20936437	-1,439704338	0	1
m_365	41435	HSV1-MIR-H8: HSV1-MIR-H8	0,0811218	0,31253233	-0,995894309	0	1
m_366	41436	HSV1-MIR-H8*: HSV1-MIR-H8*	0,1146245	0,32521372	-1,13340566	0	1
	35823	MIR-101-101C: HSA-MIR-101; MMU-MIR-101A; RNO-MIR-101A; MMU-MIR-101C			-0,003642856	0	0
	35824	MIR-10A: HSA-MIR-10A; MMU-MIR-10A; RNO-MIR-10A			0,801543477	0	0
	35826	MIR-122: HSA-MIR-122; MMU-MIR-122; RNO-MIR-122			-0,388932276	0	0
	35832	MIR-126-5P: MMU-MIR-126-5P; HSA-MIR-126*; RNO-MIR-126*			-0,452355358	0	0
	35835	MIR-129-3P: HSA-MIR-129-3P; MMU-MIR-129-2-3P; RNO-MIR-129-2*; RNO-MIR-129-1*			-0,408407329	0	0
	35836	MIR-129-5P: HSA-MIR-129-5P; MMU-MIR-129-5P; RNO-MIR-129			0,070730275	0	0
	35838	MIR-130B: HSA-MIR-130B; MMU-MIR-130B; RNO-MIR-130B			0,863808909	0	0
	35841	MIR-134: HSA-MIR-134; MMU-MIR-134; RNO-MIR-134			0,861828736	0	0
	35844	MIR-137: HSA-MIR-137; MMU-MIR-137; RNO-MIR-137			0,080091854	0	0
	35847	MIR-140-5P: HSA-MIR-140-5P; MMU-MIR-140; RNO-MIR-140			1,086060583	0	0
	35849	MIR-141: HSA-MIR-141; MMU-MIR-141; RNO-MIR-141			0,187828507	0	0
	35854	MIR-144: HSA-MIR-144; MMU-MIR-144; RNO-MIR-144			0,352417762	0	0
	35856	MIR-146A: HSA-MIR-146A; MMU-MIR-146A; RNO-MIR-146A			-0,264431651	0	0
	35857	MIR-148A: HSA-MIR-148A; MMU-MIR-148A			0,778262937	0	0
	35859	MIR-150: HSA-MIR-150; MMU-MIR-150; RNO-MIR-150			1,086659949	0	0
	35861	MIR-152: HSA-MIR-152; MMU-MIR-152; RNO-MIR-152			0,828754557	0	0
	35862	MIR-153: HSA-MIR-153; MMU-MIR-153; RNO-MIR-153			0,853198618	0	0
	35863	MIR-154: HSA-MIR-154; MMU-MIR-154; RNO-MIR-154			0,747049706	0	0
	35864	MIR-15A: HSA-MIR-15A; MMU-MIR-15A; RNO-MIR-15A			0,862228093	0	0
	35865	MIR-15B: HSA-MIR-15B; MMU-MIR-15B; RNO-MIR-15B			1,003146249	0	0
	35866	MIR-16: HSA-MIR-16; MMU-MIR-16; RNO-MIR-16			0,743143932	0	0
	35871	MIR-182_1: HSA-MIR-182; MMU-MIR-182; RNO-MIR-182			-0,069637466	0	0
	35872	MIR-187: HSA-MIR-187; MMU-MIR-187; RNO-MIR-187			0,170310319	0	0
	35876	MIR-192: HSA-MIR-192; MMU-MIR-192; RNO-MIR-192			0,160569737	0	0
	35878	MIR-194: HSA-MIR-194; MMU-MIR-194; RNO-MIR-194			0,268433086	0	0
	35882	MIR-208A: HSA-MIR-208A; MMU-MIR-208A-3P; RNO-MIR-208			-0,437578509	0	0
	35886	MIR-22: HSA-MIR-22; MMU-MIR-22; RNO-MIR-22			0,780905135	0	0
	35889	MIR-24: HSA-MIR-24; MMU-MIR-24; RNO-MIR-24			1,143711452	0	0
	35890	MIR-25: HSA-MIR-25; MMU-MIR-25; RNO-MIR-25			0,374034767	0	0
	35892	MIR-26B: HSA-MIR-26B; MMU-MIR-26B; RNO-MIR-26B			0,693757999	0	0
	35898	MIR-30A: HSA-MIR-30A; MMU-MIR-30A; RNO-MIR-30A			-0,251984283	0	0
	35901	MIR-30D: HSA-MIR-30D; MMU-MIR-30D; RNO-MIR-30D			1,156523043	0	0
	35905	MIR-324-3P: HSA-MIR-324-3P; MMU-MIR-324-3P; RNO-MIR-324-3P			0,356013696	0	0
	35912	MIR-375: HSA-MIR-375; MMU-MIR-375; RNO-MIR-375			-0,238974832	0	0
	35918	MIR-381: HSA-MIR-381; MMU-MIR-381; RNO-MIR-381			0,144720136	0	0
	35919	MIR-382: HSA-MIR-382; MMU-MIR-382; RNO-MIR-382			-0,004890306	0	0
	35926	MIR-93: HSA-MIR-93; MMU-MIR-93; RNO-MIR-93			0,849716175	0	0
	35927	MIR-96: HSA-MIR-96; MMU-MIR-96; RNO-MIR-96			0,916001768	0	0
	38223	MIR-378*: HSA-MIR-378*; MMU-MIR-378*; RNO-MIR-378*			0,392972612	0	0
	38317	MIR-103A: HSA-MIR-103A; MMU-MIR-103; RNO-MIR-103			1,184544004	0	0
	38318	MIR-107: HSA-MIR-107; MMU-MIR-107; RNO-MIR-107			1,114285336	0	0
	38319	MIR-127: HSA-MIR-127; MMU-MIR-127; RNO-MIR-127			0,587804283	0	0
	38323	MIR-181C: HSA-MIR-181C; MMU-MIR-181C; RNO-MIR-181C			0,7228578	0	0
	38324	MIR-197: HSA-MIR-197			-0,380409888	0	0
	38326	MIR-19A: HSA-MIR-19A; MMU-MIR-19A; RNO-MIR-19A			1,009329829	0	0
	38328	MIR-203: HSA-MIR-203; MMU-MIR-203; RNO-MIR-203			0,169527381	0	0
	38330	MIR-20A: HSA-MIR-20A; MMU-MIR-20A; RNO-MIR-20A			1,180438916	0	0
	38331	MIR-20B: HSA-MIR-20B; MMU-MIR-20B; RNO-MIR-20B-5P			0,142128006	0	0
	38332	MIR-211: HSA-MIR-211			0,028492431	0	0
	38333	MIR-215: HSA-MIR-215			0,634830891	0	0
	38334	MIR-218: HSA-MIR-218; MMU-MIR-218; RNO-MIR-218A			0,334763163	0	0
	38338	MIR-28-5P-28C: HSA-MIR-28-5P; MMU-MIR-28; RNO-MIR-28; MMU-MIR-28C			0,302663154	0	0
	38339	MIR-299-3P: HSA-MIR-299-3P			0,335617436	0	0
	38340	MIR-302B: HSA-MIR-302B; MMU-MIR-302B			0,074261241	0	0
	38342	MIR-302D: HSA-MIR-302D; MMU-MIR-302D			0,065298969	0	0
	38344	MIR-329: HSA-MIR-329			0,245202553	0	0
	38346	MIR-346: HSA-MIR-346			-0,146857033	0	0
	38347	MIR-362-5P: HSA-MIR-362-5P			0,852443637	0	0
	38348	MIR-363: HSA-MIR-363; MMU-MIR-363-3P; RNO-MIR-363			0,500103428	0	0
	38350	MIR-367: HSA-MIR-367; MMU-MIR-367			0,105645408	0	0

38352	MIR-369-5P: HSA-MIR-369-5P; MMU-MIR-369-5P; RNO-MIR-369-5P	0,439060672	0	0
38353	MIR-372: HSA-MIR-372	0,070953313	0	0
38354	MIR-373: HSA-MIR-373	0,129164867	0	0
38355	MIR-374A: HSA-MIR-374A	-0,227095057	0	0
38356	MIR-376A: HSA-MIR-376A	-0,11834215	0	0
38357	MIR-376B: HSA-MIR-376B	-0,079352086	0	0
38358	MIR-380-3P: HSA-MIR-380-3P	-0,042099393	0	0
38359	MIR-383: HSA-MIR-383	0,157648348	0	0
38361	MIR-411: HSA-MIR-411; MMU-MIR-411; RNO-MIR-411	0,733501425	0	0
38362	MIR-421-3P: RNO-MIR-421*; HSA-MIR-421; MMU-MIR-421	0,310676961	0	0
38363	MIR-322-MIR-424: HSA-MIR-424; (HSA-MIR-322)	0,149009382	0	0
38364	MIR-425: HSA-MIR-425; MMU-MIR-425; RNO-MIR-425	0,22197208	0	0
38367	MIR-432: HSA-MIR-432	0,481607568	0	0
38368	MIR-448: HSA-MIR-448; MMU-MIR-448-3P; RNO-MIR-448	-0,150187003	0	0
38369	MIR-449A: HSA-MIR-449A; MMU-MIR-449A; RNO-MIR-449A	0,030674863	0	0
38370	MIR-449B: HSA-MIR-449B	0,346427038	0	0
38371	MIR-455-5P: HSA-MIR-455-5P; MMU-MIR-455*; RNO-MIR-455	0,254240525	0	0
38372	MIR-484: HSA-MIR-484; MMU-MIR-484; RNO-MIR-484	-0,378392765	0	0
38374	MIR-485-5P: HSA-MIR-485-5P; MMU-MIR-485; RNO-MIR-485	-0,053552693	0	0
38375	MIR-486-5P: HSA-MIR-486-5P; MMU-MIR-486	-0,426727867	0	0
38376	MIR-487A: HSA-MIR-487A	0,225166161	0	0
38377	MIR-487B: HSA-MIR-487B; MMU-MIR-487B; RNO-MIR-487B	0,847231467	0	0
38378	MIR-492: HSA-MIR-492	-0,156586478	0	0
38380	MIR-498: HSA-MIR-498	-0,282746002	0	0
38381	MIR-500A*: HSA-MIR-500A*	0,254385309	0	0
38383	MIR-503: HSA-MIR-503	0,276838969	0	0
38384	MIR-506: HSA-MIR-506	0,285646785	0	0
38385	MIR-507: HSA-MIR-507	-0,236703714	0	0
38386	MIR-508-3P: HSA-MIR-508-3P	0,029846107	0	0
38387	MIR-511: HSA-MIR-511	0,94166256	0	0
38388	MIR-512-3P: HSA-MIR-512-3P	0,046680621	0	0
38389	MIR-515-5P: HSA-MIR-515-5P	1,083697038	0	0
38391	MIR-518B: HSA-MIR-518B	-0,224844072	0	0
38392	MIR-520A-3P: HSA-MIR-520A-3P	-0,234357154	0	0
38393	MIR-520A-5P: HSA-MIR-520A-5P	-0,058698077	0	0
38394	MIR-520G: HSA-MIR-520G	-0,138997798	0	0
38395	MIR-521: HSA-MIR-521	0,438609142	0	0
38397	MIR-525-5P: HSA-MIR-525-5P	-0,295465856	0	0
38398	MIR-539-5P: HSA-MIR-539; MMU-MIR-539-5P; RNO-MIR-539	0,302498535	0	0
38399	MIR-548A-3P: HSA-MIR-548A-3P	-0,058558469	0	0
38400	MIR-548B-3P: HSA-MIR-548B-3P	0,436563206	0	0
38401	MIR-548D-3P: HSA-MIR-548D-3P	-0,016785683	0	0
38402	MIR-549: HSA-MIR-549	-0,27981934	0	0
38403	MIR-551A: HSA-MIR-551A	0,013420707	0	0
38404	MIR-552: HSA-MIR-552	-0,144394312	0	0
38405	MIR-553: HSA-MIR-553	-0,399123484	0	0
38406	MIR-554: HSA-MIR-554	-0,167815262	0	0
38407	MIR-555: HSA-MIR-555	0,371809947	0	0
38408	MIR-557: HSA-MIR-557	0,20266196	0	0
38409	MIR-558: HSA-MIR-558	0,812952257	0	0
38410	MIR-559: HSA-MIR-559	-0,1122404	0	0
38411	MIR-561: HSA-MIR-561	-0,261242091	0	0
38414	MIR-564: HSA-MIR-564	0,17992656	0	0
38415	MIR-566: HSA-MIR-566	0,061289568	0	0
38416	MIR-567: HSA-MIR-567	0,249683892	0	0
38417	MIR-568: HSA-MIR-568; MMU-MIR-568; RNO-MIR-568	-0,146832453	0	0
38418	MIR-569: HSA-MIR-569	0,367413365	0	0
38419	MIR-571: HSA-MIR-571	-0,052418524	0	0
38421	MIR-573: HSA-MIR-573	0,233769982	0	0
38422	MIR-575: HSA-MIR-575	-0,253226292	0	0
38423	MIR-577: HSA-MIR-577	-0,228126112	0	0
38424	MIR-578: HSA-MIR-578	0,426318363	0	0
38425	MIR-580: HSA-MIR-580	0,219736944	0	0
38426	MIR-581: HSA-MIR-581	-0,103352656	0	0
38428	MIR-584: HSA-MIR-584	-0,463118558	0	0
38429	MIR-585: HSA-MIR-585	0,661260791	0	0
38430	MIR-586: HSA-MIR-586	-0,257202326	0	0
38431	MIR-587: HSA-MIR-587	0,439415353	0	0
38432	MIR-588: HSA-MIR-588	0,377675234	0	0
38433	MIR-589*: HSA-MIR-589*	-0,456424886	0	0
38434	MIR-591: HSA-MIR-591	0,644299627	0	0
38436	MIR-593*: HSA-MIR-593*	0,988524254	0	0
38437	MIR-595: HSA-MIR-595	-0,418218244	0	0
38438	MIR-596: HSA-MIR-596	-0,011400689	0	0
38439	MIR-597: HSA-MIR-597	0,291703118	0	0
38440	MIR-598-3P: HSA-MIR-598	0,713261923	0	0
38441	MIR-600: HSA-MIR-600	0,249575714	0	0
38442	MIR-601: HSA-MIR-601	-0,404167839	0	0
38444	MIR-603: HSA-MIR-603	0,138909936	0	0
38445	MIR-604: HSA-MIR-604	-0,154487037	0	0
38447	MIR-606: HSA-MIR-606	0,016491705	0	0
38448	MIR-607: HSA-MIR-607	-0,022702897	0	0
38449	MIR-608: HSA-MIR-608	-0,478169523	0	0
38450	MIR-609: HSA-MIR-609	0,235179541	0	0
38451	MIR-610: HSA-MIR-610	-0,004756558	0	0
38452	MIR-611: HSA-MIR-611	-0,086169807	0	0
38453	MIR-612: HSA-MIR-612	0,373284918	0	0
38454	MIR-613: HSA-MIR-613	0,000602836	0	0
38455	MIR-614: HSA-MIR-614	0,011807345	0	0
38456	MIR-616*: HSA-MIR-616*	0,195436928	0	0
38457	MIR-617: HSA-MIR-617	0,364187555	0	0
38458	MIR-618: HSA-MIR-618	-0,112247644	0	0
38459	MIR-619: HSA-MIR-619	0,335256553	0	0
38460	MIR-620: HSA-MIR-620	0,159767738	0	0
38461	MIR-621: HSA-MIR-621	0,039375755	0	0
38462	MIR-622: HSA-MIR-622	0,031947304	0	0
38463	MIR-623: HSA-MIR-623	-0,211171363	0	0
38464	MIR-624*: HSA-MIR-624*	0,068458184	0	0
38465	MIR-626: HSA-MIR-626	0,046544184	0	0
38466	MIR-627: HSA-MIR-627	0,588312807	0	0
38467	MIR-629*: HSA-MIR-629*	-0,02942615	0	0
38469	MIR-631: HSA-MIR-631	-0,453528506	0	0
38470	MIR-632: HSA-MIR-632	0,228966234	0	0
38471	MIR-633: HSA-MIR-633	-0,203905751	0	0
38472	MIR-634: HSA-MIR-634	-0,281713539	0	0
38473	MIR-635: HSA-MIR-635	0,385800522	0	0
38474	MIR-637: HSA-MIR-637	-0,033954192	0	0
38476	MIR-639: HSA-MIR-639	0,404053588	0	0
38477	MIR-640: HSA-MIR-640	-0,24942949	0	0
38478	MIR-641: HSA-MIR-641	0,204994706	0	0
38480	MIR-643: HSA-MIR-643	-0,004888522	0	0
38481	MIR-644: HSA-MIR-644	0,538150507	0	0
38482	MIR-645: HSA-MIR-645	0,290116968	0	0
38483	MIR-646: HSA-MIR-646	0,314236878	0	0
38484	MIR-647: HSA-MIR-647	-0,022885045	0	0
38485	MIR-648: HSA-MIR-648	0,503924895	0	0
38486	MIR-649: HSA-MIR-649	0,37543619	0	0
38487	MIR-650: HSA-MIR-650	0,014715122	0	0
38488	MIR-651: HSA-MIR-651	-0,178980043	0	0
38490	MIR-657: HSA-MIR-657	0,482677122	0	0
38494	MIR-662: HSA-MIR-662	0,467592538	0	0
38495	MIR-9: HSA-MIR-9; MMU-MIR-9; RNO-MIR-9	0,984712939	0	0
38496	MIR-95: HSA-MIR-95	0,845632731	0	0
38497	MIR-98: HSA-MIR-98; MMU-MIR-98; RNO-MIR-98	0,720142616	0	0
38498	MIR-99A: HSA-MIR-99A; MMU-MIR-99A; RNO-MIR-99A	1,1072824	0	0
38500	MIR-132: HSA-MIR-132; MMU-MIR-132; RNO-MIR-132	0,956885835	0	0
38502	MIR-184: HSA-MIR-184; MMU-MIR-184; RNO-MIR-184	-0,044279962	0	0
38505	MIR-200A: HSA-MIR-200A; MMU-MIR-200A; RNO-MIR-200A	0,210755154	0	0
38507	MIR-205: HSA-MIR-205; MMU-MIR-205; RNO-MIR-205	-0,31327563	0	0
38508	MIR-206: HSA-MIR-206; MMU-MIR-206; RNO-MIR-206	0,674617326	0	0
38519	MIR-299-5P: HSA-MIR-299-5P; MMU-MIR-299*; RNO-MIR-299	0,597937876	0	0
38521	MIR-302A: HSA-MIR-302A; MMU-MIR-302A	0,343921304	0	0
38523	MIR-31: HSA-MIR-31; MMU-MIR-31; RNO-MIR-31	0,569345793	0	0
38524	MIR-326: HSA-MIR-326; MMU-MIR-326; RNO-MIR-326	-0,360433876	0	0
38526	MIR-34C-5P: HSA-MIR-34C-5P; MMU-MIR-34C; RNO-MIR-34C	0,477836749	0	0
38531	MIR-433-3P: MMU-MIR-433 (MMU-MIR-433-3P); HSA-MIR-433; RNO-MIR-433	-0,393545961	0	0
38541	MIR-542-3P: HSA-MIR-542-3P; MMU-MIR-542-3P; RNO-MIR-542-3P	1,0791333	0	0
38542	MIR-99B: HSA-MIR-99B; MMU-MIR-99B; RNO-MIR-99B	1,061789503	0	0
38546	MIR-324-5P: HSA-MIR-324-5P; MMU-MIR-324-5P; RNO-MIR-324-5P	0,744600109	0	0
38551	MIR-331-3P: HSA-MIR-331-3P; MMU-MIR-331-3P; RNO-MIR-331	1,185733296	0	0
38553	MIR-335: HSA-MIR-335; MMU-MIR-335-5P; RNO-MIR-335	0,607224921	0	0
38562	MIR-374B-374C: HSA-MIR-374B; MMU-MIR-374; RNO-MIR-374; HSA-MIR-374C; MMU-MIR-374C	-0,166879064	0	0
38564	MIR-421-5P: HSA-MIR-421-5P; MMU-MIR-421*; RNO-MIR-421	-0,277962279	0	0
38567	MIR-499-5P: HSA-MIR-499-5P; MMU-MIR-499; RNO-MIR-499	0,248107928	0	0
38570	MIR-190: HSA-MIR-190; MMU-MIR-190; RNO-MIR-190	-0,384876402	0	0
38607	MIR-544: HSA-MIR-544	-0,442688487	0	0
38609	MIR-545: HSA-MIR-545	0,309159526	0	0
38610	MIR-548C-3P: HSA-MIR-548C-3P	-0,015259057	0	0
38611	MIR-548C-5P: HSA-MIR-548C-5P	1,120467609	0	0
38612	MIR-550A*: HSA-MIR-550A*	0,34242364	0	0
38614	MIR-551B: HSA-MIR-551B; MMU-MIR-551B; RNO-MIR-551B	0,1616915	0	0
38621	MIR-576-5P: HSA-MIR-576-5P	-0,30871163	0	0
38622	MIR-576-3P: HSA-MIR-576-3P	0,095761038	0	0
38624	MIR-579: HSA-MIR-579	0,099431114	0	0
38625	MIR-582-5P: HSA-MIR-582-5P; MMU-MIR-582-5P; RNO-MIR-582	0,899091162	0	0
38626	MIR-590-5P: HSA-MIR-590-5P; MMU-MIR-590-5P	0,661132693	0	0
38628	MIR-599: HSA-MIR-599; MMU-MIR-599	-0,482518651	0	0
38633	MIR-628-3P: HSA-MIR-628-3P	-0,029532944	0	0
38634	MIR-629: HSA-MIR-629	0,242732652	0	0
38637	MIR-652: HSA-MIR-652; MMU-MIR-652; RNO-MIR-652	0,041096392	0	0
38639	MIR-653: HSA-MIR-653; MMU-MIR-653	-0,053571277	0	0
38640	MIR-654-5P: HSA-MIR-654-5P	0,039934051	0	0
38642	MIR-660: HSA-MIR-660	1,121447486	0	0
38644	MIR-7: HSA-MIR-7; MMU-MIR-7A; RNO-MIR-7A	0,517469983	0	0
38645	MIR-92A: HSA-MIR-92A; MMU-MIR-92A; RNO-MIR-92A	1,112723716	0	0
38648	MIR-92B: HSA-MIR-92B; MMU-MIR-92B; RNO-MIR-92B	0,725495724	0	0
38652	KSHV-MIR-K12-12*: KSHV-MIR-K12-12*	-0,078177748	0	0
38653	KSHV-MIR-K12-1: KSHV-MIR-K12-1	0,218045811	0	0
38656	KSHV-MIR-K12-11: KSHV-MIR-K12-11	0,2680042	0	0
38657	KSHV-MIR-K12-2: KSHV-MIR-K12-2	-0,448849036	0	0
38659	KSHV-MIR-K12-3*: KSHV-MIR-K12-3*	0,615849314	0	0
38660	KSHV-MIR-K12-3: KSHV-MIR-K12-3	-0,470321883	0	0
38661	KSHV-MIR-K12-4-3P: KSHV-MIR-K12-4-3P	-0,294890895	0	0
38662	KSHV-MIR-K12-4-5P: KSHV-MIR-K12-4-5P	0,21558967	0	0
38663	KSHV-MIR-K12-5: KSHV-MIR-K12-5	-0,334455714	0	0
38664	KSHV-MIR-K12-6-3P: KSHV-MIR-K12-6-3P	0,355561146	0	0
38665	KSHV-MIR-K12-6-5P: KSHV-MIR-K12-6-5P	0,345890269	0	0
38666	KSHV-MIR-K12-7: KSHV-MIR-K12-7	0,04038678	0	0
38667	KSHV-MIR-K12-8: KSHV-MIR-K12-8	-0,290683962	0	0
38668	KSHV-MIR-K12-9: KSHV-MIR-K12-9	0,469023248	0	0
38679	LET-7D*: MMU-LET-7D*; RNO-LET-7D*; HSA-LET-7D*	-0,496070326	0	0
38719	MIR-186: HSA-MIR-186; MMU-MIR-186; RNO-MIR-186	-0,482833838	0	0
38723	MIR-200B: HSA-MIR-200B; MMU-MIR-200B; RNO-MIR-200B	0,346746074	0	0
38727	MIR-204: HSA-MIR-204; MMU-MIR-204; RNO-MIR-204	0,059089061	0	0
38748	MIR-297A: MMU-MIR-297A; HSA-MIR-297	0,248930546	0	0
38756	MIR-302C: HSA-MIR-302C; RNO-MIR-302C; MMU-MIR-302C	0,082456942	0	0
38789	MIR-410: HSA-MIR-410; MMU-MIR-410; RNO-MIR-410	0,272972842	0	0
38791	MIR-412: HSA-MIR-412; MMU-MIR-412-3P; RNO-MIR-412	0,34768014	0	0
38793	MIR-431: HSA-MIR-431; MMU-MIR-431; RNO-MIR-431	0,095701716	0	0
38909	MIR-720: MMU-MIR-720; HSA-MIR-720	0,23178077	0	0
38923	EBV-MIR-BART20-5P_3: EBV-MIR-BART20-5P	0,565078285	0	0
38964	MIR-140-3P: HSA-MIR-140-3P; MMU-MIR-140*; RNO-MIR-140*	0,711991895	0	0
38982	MIR-29C*: HSA-MIR-29C*; MMU-MIR-29C*; RNO-MIR-29C*	-0,009077529	0	0
38985	MIR-323-3P: HSA-MIR-323-3P; MMU-MIR-323-3P; RNO-MIR-323	-0,042614103	0	0
38986	MIR-337-3P: MMU-MIR-337-3P; RNO-MIR-337; (HSA-MIR-337-3P)	0,435123711	0	0
38989	MIR-340-3P: RNO-MIR-340-3P; MMU-MIR-340-3P; HSA-MIR-340*	0,538844954	0	0
38991	MIR-342-3P: HSA-MIR-342-3P; MMU-MIR-342-3P; RNO-MIR-342-3P	0,350858148	0	0
38998	MIR-363*: RNO-MIR-363*; HSA-MIR-363*	-0,317384764	0	0
39000	MIR-376A*: RNO-MIR-376A*; HSA-MIR-376A*; MMU-MIR-376A*	-0,167726116	0	0
39008	MIR-378: HSA-MIR-378; MMU-MIR-378; RNO-MIR-378	1,005760304	0	0
39029	MIR-7-1*: RNO-MIR-7A-1*; HSA-MIR-7-1*; MMU-MIR-7A-1*	0,384351814	0	0
39088	EBV-MIR-BART10: EBV-MIR-BART10	-0,008974463	0	0
39089	EBV-MIR-BART11-3P: EBV-MIR-BART11-3P	0,344585744	0	0
39091	EBV-MIR-BART11-5P: EBV-MIR-BART11-5P	0,158342423	0	0
39092	EBV-MIR-BART12: EBV-MIR-BART12	-0,181172778	0	0
39094	EBV-MIR-BART13: EBV-MIR-BART13	0,012320406	0	0
39095	EBV-MIR-BART14-3P: EBV-MIR-BART14-3P	-0,194563498	0	0
39098	EBV-MIR-BART15: EBV-MIR-BART15	-0,190568887	0	0
39100	EBV-MIR-BART1-5P: EBV-MIR-BART1-5P	-0,3100792	0	0
39102	EBV-MIR-BART16: EBV-MIR-BART16	0,001472773	0	0
39106	EBV-MIR-BART17-5P: EBV-MIR-BART17-5P	-0,194241984	0	0
39108	EBV-MIR-BART18-3P: EBV-MIR-BART18-3P	0,13027119	0	0
39109	EBV-MIR-BART18-5P: EBV-MIR-BART18-5P	0,687077461	0	0
39112	EBV-MIR-BART19-5P: EBV-MIR-BART19-5P	-0,420512123	0	0

39113	EBV-MIR-BART20-3P: EBV-MIR-BART20-3P	0,267975979	0	0
39116	EBV-MIR-BART2-3P: EBV-MIR-BART2-3P	-0,249761896	0	0
39117	EBV-MIR-BART2-5P: EBV-MIR-BART2-5P	0,147427729	0	0
39118	EBV-MIR-BART3-3P: EBV-MIR-BART3-3P	0,206067252	0	0
39119	EBV-MIR-BART3-5P: EBV-MIR-BART3-5P	0,200781193	0	0
39121	EBV-MIR-BART4: EBV-MIR-BART4	-0,035350241	0	0
39122	EBV-MIR-BART5: EBV-MIR-BART5	0,280731136	0	0
39125	EBV-MIR-BART6-5P: EBV-MIR-BART6-5P	0,031946044	0	0
39126	EBV-MIR-BART7: EBV-MIR-BART7	0,24378045	0	0
39129	EBV-MIR-BART8-5P: EBV-MIR-BART8-5P	0,448260111	0	0
39131	EBV-MIR-BART9: EBV-MIR-BART9	-0,458478805	0	0
39133	EBV-MIR-BHRF1-2: EBV-MIR-BHRF1-2	0,686552448	0	0
39134	EBV-MIR-BHRF1-2*: EBV-MIR-BHRF1-2*	0,813797832	0	0
39136	EBV-MIR-BHRF1-3: EBV-MIR-BHRF1-3	0,249004564	0	0
39140	HCMV-MIR-UL22A: HCMV-MIR-UL22A	0,112370738	0	0
39141	HCMV-MIR-UL22A*: HCMV-MIR-UL22A*	0,849409202	0	0
39142	HCMV-MIR-UL36: HCMV-MIR-UL36	-0,384444768	0	0
39143	HCMV-MIR-UL70-3P: HCMV-MIR-UL70-3P	-0,125435564	0	0
39144	HCMV-MIR-UL70-5P: HCMV-MIR-UL70-5P	0,207196387	0	0
39145	HCMV-MIR-US25-1: HCMV-MIR-US25-1	-0,162301861	0	0
39146	HCMV-MIR-US25-2-3P: HCMV-MIR-US25-2-3P	-0,204571302	0	0
39147	HCMV-MIR-US25-2-5P: HCMV-MIR-US25-2-5P	-0,198344747	0	0
39148	HCMV-MIR-US33-3P: HCMV-MIR-US33-3P	0,207790268	0	0
39150	HCMV-MIR-US4: HCMV-MIR-US4	-0,41538976	0	0
39151	HCMV-MIR-US5-1: HCMV-MIR-US5-1	0,343418787	0	0
39152	HCMV-MIR-US5-2: HCMV-MIR-US5-2	0,343824673	0	0
39157	LET-7I: HSA-LET-7I; MMU-LET-7I; RNO-LET-7I	0,72629785	0	0
39160	MIR-888*: HSA-MIR-888*	0,407001719	0	0
39161	MIR-888: HSA-MIR-888	1,069728907	0	0
39164	MIR-532-3P: HSA-MIR-532-3P; MMU-MIR-532-3P; RNO-MIR-532-3P	0,115946771	0	0
39168	MIR-770-3P: HSA-MIR-770-3P	0,060865851	0	0
39169	MIR-770-5P: HSA-MIR-770-5P	-0,13457411	0	0
39170	MIR-190B: HSA-MIR-190B; MMU-MIR-190B	0,106374258	0	0
39172	MIR-873: HSA-MIR-873; MMU-MIR-873; RNO-MIR-873	0,655062835	0	0
39176	MIR-885-5P: HSA-MIR-885-5P	-0,331182088	0	0
39177	MIR-590-3P: HSA-MIR-590-3P; MMU-MIR-590-3P	-0,179834511	0	0
39178	MIR-671-3P: HSA-MIR-671-3P; MMU-MIR-671-3P; RNO-MIR-671	0,474832588	0	0
39180	MIR-570: HSA-MIR-570	1,085494363	0	0
39181	MIR-708*: HSA-MIR-708*; RNO-MIR-708*; MMU-MIR-708*	0,5751645	0	0
39182	MIR-708: HSA-MIR-708; MMU-MIR-708; RNO-MIR-708	0,667730259	0	0
39184	MIR-887: HSA-MIR-887	-0,00283647	0	0
39186	MIR-574-3P: MMU-MIR-574-3P; HSA-MIR-574-3P	0,123121832	0	0
39187	MIR-574-5P: HSA-MIR-574-5P; MMU-MIR-574-5P	-0,150496574	0	0
39188	MIR-654-3P: HSA-MIR-654-3P; MMU-MIR-654-3P	0,915282535	0	0
39191	MIR-875-3P: HSA-MIR-875-3P	0,53025701	0	0
39192	MIR-875-5P: HSA-MIR-875-5P; MMU-MIR-875-5P; RNO-MIR-875	-0,088732538	0	0
39193	MIR-876-3P: HSA-MIR-876-3P	0,458964147	0	0
39194	MIR-876-5P: HSA-MIR-876-5P	-0,092655936	0	0
39198	MIR-105: HSA-MIR-105	0,495905263	0	0
39202	MIR-10B: HSA-MIR-10B; MMU-MIR-10B; RNO-MIR-10B	1,06963746	0	0
39203	MIR-758: HSA-MIR-758; MMU-MIR-758; RNO-MIR-758	0,647028329	0	0
39204	MIR-208B: HSA-MIR-208B; MMU-MIR-208B; RNO-MIR-208B	-0,424343269	0	0
39205	MIR-889: HSA-MIR-889	-0,40747465	0	0
39207	MIR-124: HSA-MIR-124; MMU-MIR-124; RNO-MIR-124	0,409335678	0	0
39208	MIR-125A-5P: HSA-MIR-125A-5P; MMU-MIR-125A-5P; RNO-MIR-125A-5P	1,055225311	0	0
39209	MIR-126-3P: HSA-MIR-126; MMU-MIR-126-3P; RNO-MIR-126	-0,262508128	0	0
39214	MIR-135B: HSA-MIR-135B; MMU-MIR-135B; RNO-MIR-135B	-0,239933693	0	0
39217	MIR-139-5P: HSA-MIR-139-5P; MMU-MIR-139-5P; RNO-MIR-139-5P	0,211119411	0	0
39219	MIR-142-5P: HSA-MIR-142-5P; MMU-MIR-142-5P; RNO-MIR-142-5P	0,001637308	0	0
39224	MIR-147A: HSA-MIR-147A (HSA-MIR-147)	0,424778863	0	0
39225	MIR-147B: HSA-MIR-147B; MMU-MIR-147; RNO-MIR-147	0,817253992	0	0
39226	MIR-149: HSA-MIR-149; MMU-MIR-149; RNO-MIR-149-5P	-0,142379697	0	0
39227	MIR-151-3P: HSA-MIR-151-3P	0,814640332	0	0
39232	MIR-154*: HSA-MIR-154*; MMU-MIR-154*	0,775056235	0	0
39234	MIR-155: HSA-MIR-155	-0,050791614	0	0
39235	MIR-17*: HSA-MIR-17*; RNO-MIR-17-1-3P	0,192040841	0	0
39238	MIR-181D: HSA-MIR-181D; MMU-MIR-181D; RNO-MIR-181D	1,194998578	0	0
39240	MIR-182*: HSA-MIR-182*; MMU-MIR-182*	0,711436137	0	0
39246	MIR-188-3P: HSA-MIR-188-3P; MMU-MIR-188-3P; RNO-MIR-188*	0,188522467	0	0
39247	MIR-188-5P: HSA-MIR-188-5P; MMU-MIR-188-5P; RNO-MIR-188	0,419523471	0	0
39248	MIR-24-1*: HSA-MIR-24-1*; MMU-MIR-24-1*; RNO-MIR-24-1*	0,648055066	0	0
39251	MIR-18A: HSA-MIR-18A; MMU-MIR-18A; RNO-MIR-18A	1,013419326	0	0
39253	MIR-18B: HSA-MIR-18B	0,667855424	0	0
39255	MIR-191: HSA-MIR-191; MMU-MIR-191; RNO-MIR-191	0,424573958	0	0
39257	MIR-193B: HSA-MIR-193B	0,237663164	0	0
39262	MIR-196B: HSA-MIR-196B; MMU-MIR-196B; RNO-MIR-196B	0,554672947	0	0
39267	MIR-890: HSA-MIR-890	-0,209049347	0	0
39268	MIR-891B: HSA-MIR-891B	0,570528451	0	0
39269	MIR-877: HSA-MIR-877; MMU-MIR-877; RNO-MIR-877	-0,458314596	0	0
39271	MIR-200A*: HSA-MIR-200A*; MMU-MIR-200A*; RNO-MIR-200A*	0,107224207	0	0
39273	MIR-200C: HSA-MIR-200C; MMU-MIR-200C; RNO-MIR-200C	0,182347279	0	0
39274	MIR-202-3P: HSA-MIR-202	-0,162699265	0	0
39275	MIR-202-5P: HSA-MIR-202*; MMU-MIR-202-5P; RNO-MIR-202	-0,004535788	0	0
39282	MIR-216A: HSA-MIR-216A; MMU-MIR-216A; RNO-MIR-216A	0,233466466	0	0
39283	MIR-216B: HSA-MIR-216B; MMU-MIR-216B; RNO-MIR-216B	0,058160032	0	0
39284	MIR-217: HSA-MIR-217	0,040760896	0	0
39292	MIR-224: HSA-MIR-224; RNO-MIR-224	0,715922907	0	0
39296	MIR-28-3P: HSA-MIR-28-3P; RNO-MIR-28*	0,184340447	0	0
39300	MIR-29C: HSA-MIR-29C; MMU-MIR-29C; RNO-MIR-29C	0,334415807	0	0
39301	MIR-300-3P: HSA-MIR-300	0,124737999	0	0
39302	MIR-301B: HSA-MIR-301B	0,519336418	0	0
39303	MIR-302A*: HSA-MIR-302A*	0,790111186	0	0
39304	MIR-302B*: HSA-MIR-302B*	0,996421488	0	0
39306	MIR-30A*: HSA-MIR-30A*; MMU-MIR-30A*; RNO-MIR-30A*	-0,059669081	0	0
39308	MIR-30E*: HSA-MIR-30E*; MMU-MIR-30E*; RNO-MIR-30E*	0,958334535	0	0
39312	MIR-320A: HSA-MIR-320A; MMU-MIR-320; RNO-MIR-320	0,966627298	0	0
39315	MIR-323-5P: HSA-MIR-323-5P; MMU-MIR-323-5P; RNO-MIR-323*	0,37798514	0	0
39317	MIR-325-3P: HSA-MIR-325-3P	-0,322895165	0	0
39320	MIR-330-5P: HSA-MIR-330-5P; MMU-MIR-330; RNO-MIR-330-5P	0,002989846	0	0
39321	MIR-331-5P: HSA-MIR-331-5P; MMU-MIR-331-5P; RNO-MIR-331-5P	-0,065878564	0	0
39323	MIR-337-3P: HSA-MIR-337-3P; (RNO-MIR-337); (MMU-MIR-337-3P)	0,168232533	0	0
39324	MIR-337-5P: HSA-MIR-337-5P	0,90962703	0	0
39326	MIR-339-3P: HSA-MIR-339-3P	0,370711665	0	0
39327	MIR-339-5P: HSA-MIR-339-5P; MMU-MIR-339-5P; RNO-MIR-339-5P	0,820387971	0	0
39328	MIR-33A: MMU-MIR-33; RNO-MIR-33; HSA-MIR-33A	-0,261918761	0	0
39329	MIR-33B: HSA-MIR-33B	-0,474212697	0	0
39331	MIR-340-5P: HSA-MIR-340; MMU-MIR-340-5P; RNO-MIR-340-5P	-0,373533042	0	0
39334	MIR-345-5P: HSA-MIR-345	0,206712662	0	0
39338	MIR-34B-5P: HSA-MIR-34B*	0,625119342	0	0
39339	MIR-362-3P: HSA-MIR-362-3P	1,160368171	0	0
39340	MIR-582-3P: HSA-MIR-582-3P	0,093210837	0	0
39345	MIR-371-3P: HSA-MIR-371-3P	-0,296945049	0	0
39351	MIR-380-5P: HSA-MIR-380*; MMU-MIR-380-5P; RNO-MIR-380	0,467660449	0	0
39358	MIR-409-5P: HSA-MIR-409-5P; MMU-MIR-409-5P; RNO-MIR-409-5P	0,086384091	0	0
39361	MIR-422A: HSA-MIR-422A	0,305373361	0	0
39364	MIR-423-3P: HSA-MIR-423-3P; MMU-MIR-423-3P; RNO-MIR-423	0,460242894	0	0
39365	MIR-423-5P: HSA-MIR-423-5P; MMU-MIR-423-5P; RNO-MIR-423*	-0,380439751	0	0
39366	MIR-432*: HSA-MIR-432*	0,054782412	0	0
39368	MIR-450A-5P: HSA-MIR-450A; MMU-MIR-450A; RNO-MIR-450A-2	-0,39928519	0	0
39370	MIR-451: HSA-MIR-451; MMU-MIR-451; RNO-MIR-451	-0,185991479	0	0
39373	MIR-452: HSA-MIR-452; MMU-MIR-452-5P	0,045041351	0	0
39375	MIR-323B-5P: HSA-MIR-323B-5P	0,35384356	0	0
39378	MIR-802: MMU-MIR-802; HSA-MIR-802; RNO-MIR-802	-0,074587272	0	0
39379	MIR-892A: HSA-MIR-892A	0,263061335	0	0
39383	MIR-488*: HSA-MIR-488*	0,187648339	0	0
39384	MIR-488: HSA-MIR-488	0,904704605	0	0
39385	MIR-489: HSA-MIR-489	0,325170065	0	0
39387	MIR-490-3P: HSA-MIR-490-3P; MMU-MIR-490; RNO-MIR-490	0,379026371	0	0
39388	MIR-490-5P: HSA-MIR-490-5P; MMU-MIR-490-5P; RNO-MIR-490-5P	1,193590321	0	0
39390	MIR-491-3P: HSA-MIR-491-3P; MMU-MIR-491-3P	0,500685902	0	0
39392	MIR-493: RNO-MIR-493; HSA-MIR-493	0,363493683	0	0
39394	MIR-494: HSA-MIR-494; MMU-MIR-494; RNO-MIR-494	-0,183070493	0	0
39396	MIR-495: HSA-MIR-495; MMU-MIR-495; RNO-MIR-495	0,138000749	0	0
39400	MIR-501-3P: HSA-MIR-501-3P	0,239959184	0	0
39401	MIR-502-5P: HSA-MIR-502-5P	-0,054138681	0	0
39402	MIR-502-3P: HSA-MIR-502-3P	0,459213133	0	0
39404	MIR-504: HSA-MIR-504; MMU-MIR-504	0,067326354	0	0
39406	MIR-505: HSA-MIR-505; RNO-MIR-505	0,75256081	0	0
39408	MIR-509-3P: HSA-MIR-509-3P	-0,140697968	0	0
39409	MIR-509-5P: HSA-MIR-509-5P	-0,012252119	0	0
39410	MIR-509-3-5P: HSA-MIR-509-3-5P	0,228309648	0	0
39412	MIR-510: HSA-MIR-510	0,09792804	0	0
39414	MIR-512-5P: HSA-MIR-512-5P	0,112846686	0	0
39415	MIR-513A-5P: HSA-MIR-513A-5P	-0,356615599	0	0
39416	MIR-513A-3P: HSA-MIR-513A-3P	-0,049749047	0	0
39419	MIR-514: HSA-MIR-514	0,80599165	0	0
39421	MIR-515-3P: HSA-MIR-515-3P	0,072682208	0	0
39422	MIR-516A-3P-MIR-516*: HSA-MIR-516A-3P; HSA-MIR-516B*	0,09084079	0	0
39423	MIR-516B: HSA-MIR-516B	-0,195280183	0	0
39424	MIR-516A-5P: HSA-MIR-516A-5P	-0,210910845	0	0
39426	MIR-517*: HSA-MIR-517*	0,153961476	0	0
39427	MIR-517A-MIR-517B_1: HSA-MIR-517A; HSA-MIR-517B	0,062376911	0	0
39433	MIR-518A-3P: HSA-MIR-518A-3P	0,054493092	0	0
39436	MIR-518C: HSA-MIR-518C	-0,456591813	0	0
39438	MIR-518D-5P-520C-5P-526A: HSA-MIR-518D-5P; HSA-MIR-520C-5P; HSA-MIR-526A	-0,290042618	0	0
39439	MIR-518D-3P: HSA-MIR-518D-3P	0,754209907	0	0
39440	MIR-518E: HSA-MIR-518E	0,741012493	0	0
39441	MIR-518E*-519A*-1-519B-5P-519C-5P-522*-523*-526C: HSA-MIR-518E*; HSA-MIR-519A*; HSA-MIR-519B-5P; HSA-MIR-519C-5P; HSA-MIR-522*;	-0,219854887	0	0
39444	MIR-518F*: HSA-MIR-518F*	0,115135998	0	0
39446	MIR-519A: HSA-MIR-519A	1,051034103	0	0
39448	MIR-519B-3P: HSA-MIR-519B-3P	0,677201196	0	0
39450	MIR-519C-3P: HSA-MIR-519C-3P	-0,160395819	0	0
39451	MIR-519D: HSA-MIR-519D	0,204899033	0	0
39455	MIR-519E: HSA-MIR-519E	0,664874744	0	0
39456	MIR-520C-3P-520F-520B_4: HSA-MIR-520C-3P; HSA-MIR-520F; HSA-MIR-520B	0,405160171	0	0
39460	MIR-520D-3P: HSA-MIR-520D-3P	-0,019366724	0	0
39464	MIR-520E: HSA-MIR-520E	0,846603741	0	0
39467	MIR-520H: HSA-MIR-520H	0,628406475	0	0
39468	MIR-522: HSA-MIR-522	1,109725962	0	0
39471	MIR-523: HSA-MIR-523	-0,332379743	0	0
39472	MIR-524-3P: HSA-MIR-524-3P	-0,21715081	0	0
39474	MIR-525-3P: HSA-MIR-525-3P	0,040577323	0	0
39477	MIR-526B*: HSA-MIR-526B*	0,598995515	0	0
39479	MIR-519C-5P: HSA-MIR-519C-5P	-0,390500705	0	0
39481	MIR-532-5P: HSA-MIR-532-5P; MMU-MIR-532-5P	0,469127471	0	0
39482	MIR-541-5P: HSA-MIR-541*	-0,223482909	0	0
39484	MIR-542-5P: HSA-MIR-542-5P; MMU-MIR-542-5P; RNO-MIR-542-5P	-0,172579548	0	0
39485	MIR-543-3P: HSA-MIR-543; MMU-MIR-543; RNO-MIR-543*	-0,14630389	0	0
39793	MIR-501-5P: HSA-MIR-501-5P; RNO-MIR-501	0,312546194	0	0
39859	EBV-MIR-BART1-3P: EBV-MIR-BART1-3P	0,025310606	0	0
39864	MIR-592: HSA-MIR-592; MMU-MIR-592; RNO-MIR-592	0,34945112	0	0
39865	MIR-671-5P: HSA-MIR-671-5P; MMU-MIR-671-5P; RNO-MIR-671-5P	-0,231735412	0	0
39866	MIR-668: HSA-MIR-668; MMU-MIR-668; RNO-MIR-668	0,117380951	0	0
39870	MIR-496_2: HSA-MIR-496; MMU-MIR-496; RNO-MIR-496	0,285579563	0	0
39877	MIR-761: MMU-MIR-761; HSA-MIR-761; RNO-MIR-761	0,276646602	0	0
39880	MIR-759: MMU-MIR-759; HSA-MIR-759; RNO-MIR-759	0,773236562	0	0
39881	MIR-760-3P: HSA-MIR-760; MMU-MIR-760-3P; RNO-MIR-760-3P	-0,404657726	0	0
39887	MIR-767-5P: HSA-MIR-767-5P; MMU-MIR-767	0,112759723	0	0
39888	MIR-767-3P: HSA-MIR-767-3P	-0,236691068	0	0
39889	MIR-454*: HSA-MIR-454*	0,814956528	0	0
39890	MIR-454_2: HSA-MIR-454	0,33459456	0	0
39891	MIR-769-5P: HSA-MIR-769-5P	0,413778441	0	0
39893	MIR-766: HSA-MIR-766	0,024008883	0	0
39898	MIR-500A-500B: HSA-MIR-500A; HSA-MIR-500B	0,808280281	0	0
39905	SV40-MIR-S1-3P: SV40-MIR-S1-3P	0,23949851	0	0
39912	HIV1-MIR-H1: HIV1-MIR-H1	-0,398661642	0	0
39913	HIV1-MIR-N367: HIV1-MIR-N367	0,123972284	0	0
39935	MIR-548D-5P: HSA-MIR-548D-5P	0,060545293	0	0
39937	MIR-148A*: HSA-MIR-148A*; MMU-MIR-148A*	-0,49638082	0	0
39938	MIR-944: HSA-MIR-944	-0,494564446	0	0
39940	MIR-499-3P: HSA-MIR-499-3P; MMU-MIR-499*	0,065931477	0	0
39942	MIR-181C*: HSA-MIR-181C*; MMU-MIR-181C*; RNO-MIR-181C*	-0,264359914	0	0
39945	MIR-129*: HSA-MIR-129*; RNO-MIR-129-1*	-0,074379153	0	0
39953	MIR-96*: HSA-MIR-96*; RNO-MIR-96*	-0,04422381	0	0
39956	MIR-10B*: HSA-MIR-10B*; MMU-MIR-10B*; RNO-MIR-10B*	-0,365120737	0	0
39959	MIR-181A-2*: HSA-MIR-181A-2*	0,697616292	0	0
39962	MIR-132*: HSA-MIR-132*; MMU-MIR-132*	-0,042767216	0	0
39963	MIR-221*: HSA-MIR-221*; MMU-MIR-221*; RNO-MIR-221*	-0,276408626	0	0
39964	MIR-105*: HSA-MIR-105*	-0,412476919	0	0
39970	MIR-130B*: MMU-MIR-130B*; HSA-MIR-130B*	-0,372432525	0	0
39971	MIR-29A*: HSA-MIR-29A*; MMU-MIR-29A*; RNO-MIR-29A*	0,239532494	0	0
39974	MIR-20A*: HSA-MIR-20A*	-0,02982956	0	0
39975	MIR-93*: HSA-MIR-93*; MMU-MIR-93*; RNO-MIR-93*	-0,179125969	0	0
39976	LET-7G*: MMU-LET-7G*; HSA-LET-7G*	0,016592295	0	0

39977	MIR-20B*: HSA-MIR-20B*	-0,059921892	0	0
39978	MIR-367*: HSA-MIR-367*	-0,046528129	0	0
39980	MIR-302D*: HSA-MIR-302D*; MMU-MIR-302D*	-0,470139521	0	0
39983	MIR-377*: HSA-MIR-377*	-0,211305423	0	0
39984	MIR-924: HSA-MIR-924	-0,291198828	0	0
39991	MIR-27A*: HSA-MIR-27A*; MMU-MIR-27A*; RNO-MIR-27A*	-0,127245553	0	0
39996	MIR-92A-1*: HSA-MIR-92A-1*	-0,288896679	0	0
39997	MIR-616: HSA-MIR-616	-0,074556714	0	0
39999	MIR-19B-1*: HSA-MIR-19B-1*; RNO-MIR-19B-1*	-0,148904663	0	0
40000	MIR-19B-2*: HSA-MIR-19B-2*	-0,056065063	0	0
40001	MIR-19A*: HSA-MIR-19A*; MMU-MIR-19A*	-0,075058648	0	0
40004	MIR-556-3P: HSA-MIR-556-3P	-0,408725856	0	0
40009	MIR-135B*: HSA-MIR-135B*	0,340420776	0	0
40013	MIR-497*: HSA-MIR-497*; MMU-MIR-497*; RNO-MIR-497*	-0,305387965	0	0
40014	MIR-10A*: HSA-MIR-10A*; MMU-MIR-10A*; RNO-MIR-10A-3P	0,031387999	0	0
40015	MIR-7-2*: HSA-MIR-7-2*	-0,44006471	0	0
40016	MIR-21*: HSA-MIR-21*	0,277586647	0	0
40019	MIR-99B*: HSA-MIR-99B*; MMU-MIR-99B*; RNO-MIR-99B*	-0,386098991	0	0
40021	MIR-100*: HSA-MIR-100*	0,183555381	0	0
40023	MIR-34A*: HSA-MIR-34A*; MMU-MIR-34A*; RNO-MIR-34A*	-0,399543917	0	0
40024	MIR-33*: HSA-MIR-33A*; MMU-MIR-33*; RNO-MIR-33*	-0,221966588	0	0
40025	MIR-32*: HSA-MIR-32*; MMU-MIR-32*; RNO-MIR-32*	-0,457356158	0	0
40026	MIR-941: HSA-MIR-941	-0,249888651	0	0
40030	MIR-15A*: HSA-MIR-15A*	-0,424717921	0	0
40031	MIR-431*: HSA-MIR-431*; MMU-MIR-431*	0,039879405	0	0
40033	MIR-101*: HSA-MIR-101*	-0,16559071	0	0
40035	MIR-136*: HSA-MIR-136*; RNO-MIR-136*; MMU-MIR-136*	-0,028530828	0	0
40036	MIR-200B*: HSA-MIR-200B*; MMU-MIR-200B*	-0,260908812	0	0
40037	MIR-141*: HSA-MIR-141*	-0,287186973	0	0
40042	MIR-16-1*: HSA-MIR-16-1*	-0,408501113	0	0
40049	MIR-106B*: HSA-MIR-106B*; MMU-MIR-106B*; RNO-MIR-106B*	0,524923073	0	0
40055	EBV-MIR-BART7*: EBV-MIR-BART7*	-0,343400798	0	0
40056	MIR-26B*: HSA-MIR-26B*; MMU-MIR-26B*; RNO-MIR-26B*	-0,414821489	0	0
40062	MIR-196A*: HSA-MIR-196A*; MMU-MIR-196A-2*; RNO-MIR-196A*	-0,300782381	0	0
40065	MIR-200C*: HSA-MIR-200C*; MMU-MIR-200C*; RNO-MIR-200C*	0,188699155	0	0
40066	MIR-223*: HSA-MIR-223*; MMU-MIR-223*; RNO-MIR-223*	-0,45055507	0	0
40070	LET-7A*-LET-7C-2*: MMU-LET-7A-1*; MMU-LET-7C-2*; HSA-LET-7A*	-0,414958201	0	0
40071	LET-7F-1*: MMU-LET-7F-1*; HSA-LET-7F-1*; RNO-LET-7F-1*	-0,498327279	0	0
40072	LET-7F-2*: HSA-LET-7F-2*; MMU-LET-7F-2*; RNO-LET-7F-2*	-0,289672684	0	0
40075	MIR-222*: HSA-MIR-222*; MMU-MIR-222*; RNO-MIR-222*	-0,127690855	0	0
40076	MIR-452*: HSA-MIR-452*	0,178981382	0	0
40077	MIR-155*: HSA-MIR-155*	-0,043804107	0	0
40080	MIR-943: HSA-MIR-943	-0,438237603	0	0
40081	MIR-106A*: HSA-MIR-106A*	-0,305454541	0	0
40082	MIR-192*: HSA-MIR-192*	-0,271855512	0	0
40083	LET-7I*: HSA-LET-7I*; MMU-LET-7I*; RNO-LET-7I*	0,204598689	0	0
40090	MIR-29B-2*: HSA-MIR-29B-2*; RNO-MIR-29B-2*; MMU-MIR-29B-2*	-0,389693659	0	0
40093	MIR-744*: HSA-MIR-744*; MMU-MIR-744*	-0,294677666	0	0
40094	MIR-374B*-374C*: HSA-MIR-374B*; MMU-MIR-374C*	-0,418423335	0	0
40096	MIR-30D*: HSA-MIR-30D*; RNO-MIR-30D*	-0,151359363	0	0
40100	MIR-922: HSA-MIR-922	-0,161145138	0	0
40101	EBV-MIR-BART10*: EBV-MIR-BART10*	-0,420267286	0	0
40102	MIR-186*: HSA-MIR-186*; RNO-MIR-186*	-0,242459577	0	0
40107	MIR-138-2*: HSA-MIR-138-2*; MMU-MIR-138-2*; RNO-MIR-138-2*	0,480264466	0	0
40111	MIR-29B-1*: HSA-MIR-29B-1*; MMU-MIR-29B-1*	0,243593617	0	0
40115	MIR-145*: HSA-MIR-145*; MMU-MIR-145*; RNO-MIR-145*	-0,12783571	0	0
40119	MIR-920: HSA-MIR-920	-0,349363677	0	0
40133	MIR-411*: HSA-MIR-411*; MMU-MIR-411*; RNO-MIR-411*	0,107440701	0	0
40140	HCMV-MIR-US25-1*: HCMV-MIR-US25-1*	-0,242281354	0	0
40145	MIR-589: HSA-MIR-589	-0,168596003	0	0
40149	MIR-24-2*: HSA-MIR-24-2*	-0,110979218	0	0
40150	MIR-214*: HSA-MIR-214*; MMU-MIR-214*	0,385586654	0	0
40152	MIR-31*: MMU-MIR-31*; HSA-MIR-31*	-0,207176214	0	0
40154	HSV1-MIR-H1: HSV1-MIR-H1	-0,361281928	0	0
40161	MIR-541-3P: HSA-MIR-541	-0,067873542	0	0
40196	MIR-182_2: HSA-MIR-182 (ALTERNATIVE MATURE SEQUENCE)	-0,298688128	0	0
40220	MIR-1227: HSA-MIR-1227	-0,419260666	0	0
40224	MIR-1231: HSA-MIR-1231	-0,442502931	0	0
40229	MIR-1238: HSA-MIR-1238	-0,290323335	0	0
40230	MIR-513B: HSA-MIR-513B	-0,301634176	0	0
40287	MIR-1178: HSA-MIR-1178	-0,278429529	0	0
40288	MIR-1179: HSA-MIR-1179	-0,436691124	0	0
40319	MIR-1244: HSA-MIR-1244	-0,353877411	0	0
40321	MIR-1246: HSA-MIR-1246	-0,199304121	0	0
40326	MIR-1251: HSA-MIR-1251	0,343613174	0	0
40330	MIR-1255A: HSA-MIR-1255A	-0,322634481	0	0
40331	MIR-1255B: HSA-MIR-1255B	-0,383151224	0	0
40336	MIR-1260: HSA-MIR-1260	0,335502928	0	0
40337	MIR-1261: HSA-MIR-1261	0,189726613	0	0
40338	MIR-1262: HSA-MIR-1262	-0,373231606	0	0
40339	MIR-1263: HSA-MIR-1263	0,053637814	0	0
40340	MIR-1264: HSA-MIR-1264	-0,475900767	0	0
40342	MIR-1266: HSA-MIR-1266	0,469580732	0	0
40345	MIR-1269: HSA-MIR-1269	-0,450031171	0	0
40347	MIR-1271: HSA-MIR-1271	-0,196537218	0	0
40357	MIR-1280: HSA-MIR-1280	-0,387166459	0	0
40364	MIR-1287: HSA-MIR-1287	0,208302874	0	0
40367	MIR-1290: HSA-MIR-1290	-0,381831302	0	0
40374	MIR-1297: HSA-MIR-1297	-0,090758987	0	0
40375	MIR-1298: HSA-MIR-1298	-0,322300429	0	0
40380	MIR-1302: HSA-MIR-1302	-0,398172051	0	0
40384	MIR-1306-3P: HSA-MIR-1306; MMU-MIR-1306-3P	-0,25160611	0	0
40388	MIR-1322: HSA-MIR-1322	0,018556784	0	0
40394	MIR-302E: HSA-MIR-302E	-0,300746836	0	0
40396	MIR-320B: HSA-MIR-320B	0,971302627	0	0
40397	MIR-320C: HSA-MIR-320C	1,157342531	0	0
40404	MIR-548E: HSA-MIR-548E	-0,492344472	0	0
40406	MIR-548H: HSA-MIR-548H	-0,431073146	0	0
40407	MIR-548I: HSA-MIR-548I	-0,2859304	0	0
40408	MIR-548J: HSA-MIR-548J	-0,416445656	0	0
40409	MIR-548K: HSA-MIR-548K	-0,3562176	0	0
40410	MIR-548L: HSA-MIR-548L	-0,434043537	0	0
40411	MIR-548M: HSA-MIR-548M	-0,157127714	0	0
40415	MIR-663B: HSA-MIR-663B	-0,332595832	0	0
40417	MIR-664-5P: HSA-MIR-664*	-0,393526405	0	0
40428	MIR-1468: HSA-MIR-1468	-0,402828366	0	0
40432	MIR-1537: HSA-MIR-1537	-0,378773124	0	0
40439	MIR-1911: HSA-MIR-1911	-0,300795092	0	0
40441	MIR-1912: HSA-MIR-1912	-0,290220381	0	0
40468	HSV1-MIR-H3: HSV1-MIR-H3	-0,333076476	0	0
40469	HSV1-MIR-H4: HSV1-MIR-H4	-0,278298607	0	0
40470	HSV1-MIR-H4*: HSV1-MIR-H4*	-0,347591386	0	0
40471	HSV1-MIR-H5-3P: HSV1-MIR-H5-3P	-0,464107114	0	0
40474	JCV-MIR-J1-3P-BKV-MIR-B1-3P: JCV-MIR-J1-3P; BKV-MIR-B1-3P	-0,45481621	0	0
40478	MIR-196B*: HSA-MIR-196B*; RNO-MIR-196B*	-0,102696969	0	0
40479	MIR-1972: HSA-MIR-1972	-0,105411791	0	0
40480	MIR-1973: HSA-MIR-1973	0,82215697	0	0
40483	MIR-1976: HSA-MIR-1976	0,388443573	0	0
40487	MIR-205*: HSA-MIR-205*	-0,081632921	0	0
40488	MIR-2052: HSA-MIR-2052	-0,178030262	0	0
40489	MIR-2053: HSA-MIR-2053	-0,485176907	0	0
40495	MIR-449B*: HSA-MIR-449B*	0,20362856	0	0
40558	EBV-MIR-BART4*: EBV-MIR-BART4*	-0,030010857	0	0
40562	HSV2-MIR-H4-3P: HSV2-MIR-H4-3P	-0,119107239	0	0
40563	HSV2-MIR-H4-5P: HSV2-MIR-H4-5P	-0,403208148	0	0
41415	MIR-2114: HSA-MIR-2114	-0,311213503	0	0
41420	MIR-2116*: HSA-MIR-2116*	-0,4606188	0	0
41421	MIR-2117: HSA-MIR-2117	-0,260293881	0	0
41422	MIR-2276: HSA-MIR-2276	-0,498440704	0	0
41423	MIR-2277-3P: HSA-MIR-2277-3P	-0,46061163	0	0
41425	MIR-449C: HSA-MIR-449C	0,004983667	0	0
41426	MIR-449C*: HSA-MIR-449C*	-0,374102568	0	0
41427	MIR-548Q: HSA-MIR-548Q	-0,427609648	0	0
41428	MIR-670: HSA-MIR-670	0,38611387	0	0
41432	MIR-764: HSA-MIR-764	-0,378000676	0	0
